# Schiff base chemosensors for neutral analytes: optical transduction mechanisms, analytical performance, and application prospects

**DOI:** 10.1039/d6ra03902b

**Published:** 2026-08-17

**Authors:** Barkha Rathee, Garima Rathee, Parul Bhalla

**Affiliations:** a Department of Chemistry, MDU Rohtak-124001 Haryana India barkha.rp.chem@mdurohtak.ac.in; b Department of Chemical Engineering, Universitat Politècnica de Catalunya 08222 Terrassa Spain; c Dr BansiDhar Institute Sector 18 Gurugram Haryana-122015 India; d St. Andrews Institute of Technology and Management Gurugram Haryana India

## Abstract

The selective optical detection of neutral organic and biologically relevant analytes remains a central challenge in analytical chemistry, as the absence of formal charge and structural diversity among targets render conventional ion-responsive sensing frameworks largely inadequate. Schiff base-derived chemosensors, defined by their synthetically accessible azomethine (–C=N–) core, have emerged as particularly well-suited molecular scaffolds for addressing this gap, owing to their modular structural tunability, straightforward synthesis from commercially available precursors, and capacity to support diverse photophysical transduction mechanisms. While early investigations centred predominantly on transition and heavy metal ion detection, research over the past decade has decisively expanded the scope of Schiff base probes toward neutral analytes of biological, environmental, and security relevance. This review critically evaluates peer-reviewed studies (2000–2025) reporting Schiff base-derived colorimetric and fluorescent probes for the detection of amino acids, biothiols, reactive selenium species, vitamins, hydrazine, thiophenols, pesticides, herbicides, nitroaromatic explosives, formaldehyde, volatile organic compounds, and selected inorganic neutral molecules. Sensing mechanisms, including photoinduced electron transfer (PET), intramolecular charge transfer (ICT), chelation-enhanced fluorescence (CHEF), excited-state intramolecular proton transfer (ESIPT), Förster resonance energy transfer (FRET), and aggregation-induced emission enhancement (AIEE), are analyzed in direct correlation with molecular design strategies and structure–property relationships. A comparative synthesis of key analytical figures of merit, encompassing detection limits (reaching sub-nanomolar levels in selected systems), selectivity profiles, response kinetics, solvent compatibility, reversibility, and real-sample applicability, reveals marked progress in probe sophistication and practical performance. Nonetheless, critical limitations persist, including susceptibility to imine hydrolysis under physiological and aqueous conditions, inadequate water solubility, and predominantly irreversible binding modes. Future development should prioritise computationally guided probe design, nanomaterial-integrated architectures, and miniaturised solid-state platforms to advance Schiff base chemosensors toward translational deployment in clinical diagnostics, environmental surveillance, food safety screening, and live-cell bioimaging.

## Abstract 2

Schiff-base chemosensors: colorimetric and fluorometric analyte detection for clinical, environmental, food safety and security applications.

## Graphical abstract

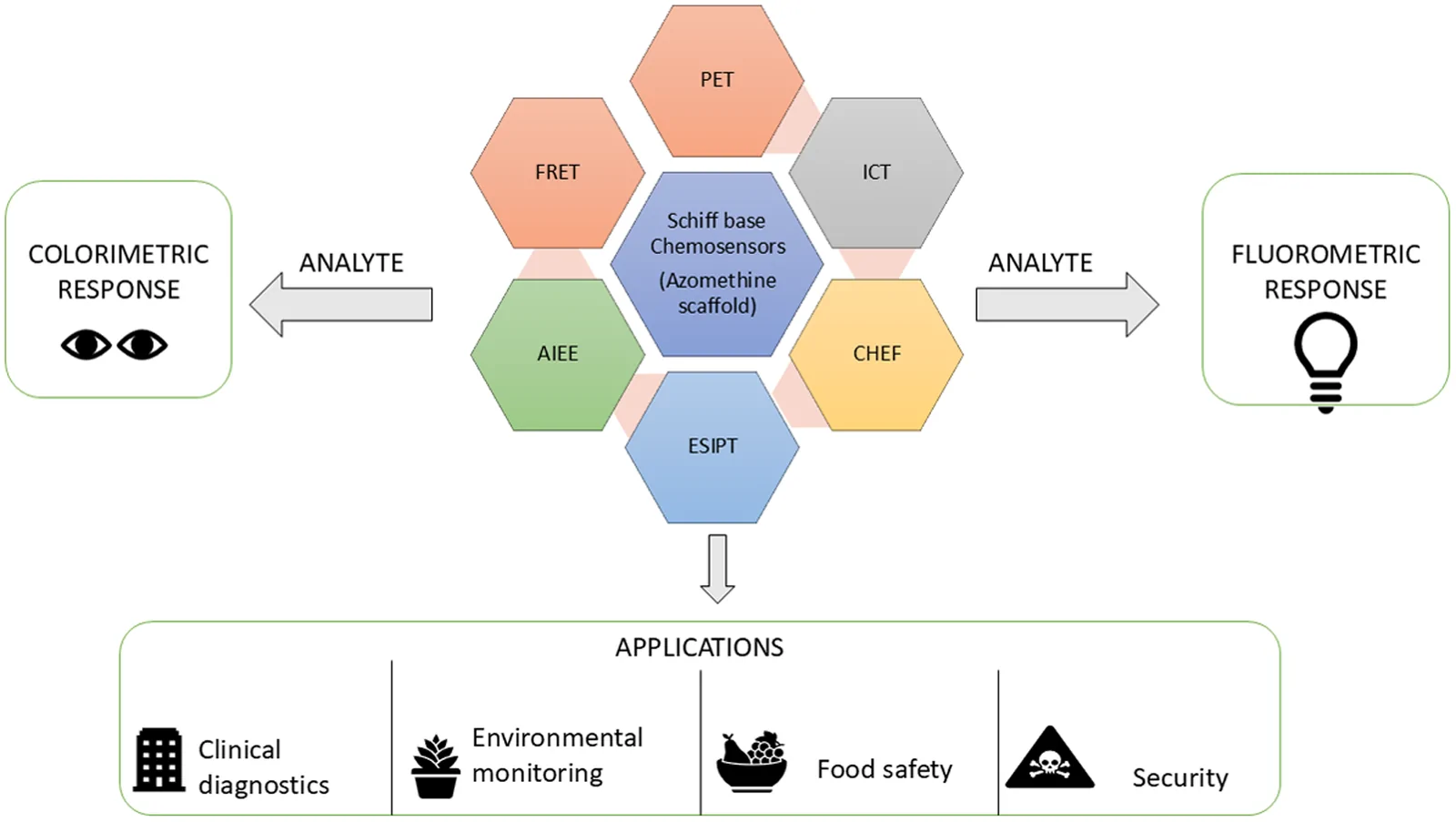

## 1 Introduction

The selective and sensitive detection of chemically and biologically relevant analytes in complex matrices remains a central challenge in analytical chemistry, environmental monitoring, and clinical diagnostics. Optical chemosensors, encompassing both fluorescent and colorimetric molecular probes, have emerged as particularly powerful analytical tools on account of their operational simplicity, high sensitivity, real-time response capability, and compatibility with biological systems.^1–3^ A chemosensor is a molecular device designed to bind a target analyte selectively and transduce this recognition event into a measurable optical or electrochemical signal. Structurally, it comprises three integrated components: a receptor (recognition unit) that provides selective analyte binding, a spacer (transducer) that communicates the binding event, and a reporter (signalling unit) that generates the detectable output.^4–6^ The field has expanded rapidly over the past two decades. A survey of the literature shows that publications on fluorescent chemosensors have increased from a few hundred annually in the early 2000s to well over two thousand per year by 2024. This growth reflects the increasing demand for portable, cost-effective, and selective detection platforms for environmental, biomedical, and food safety applications.^7,8^

**Fig. 1:**
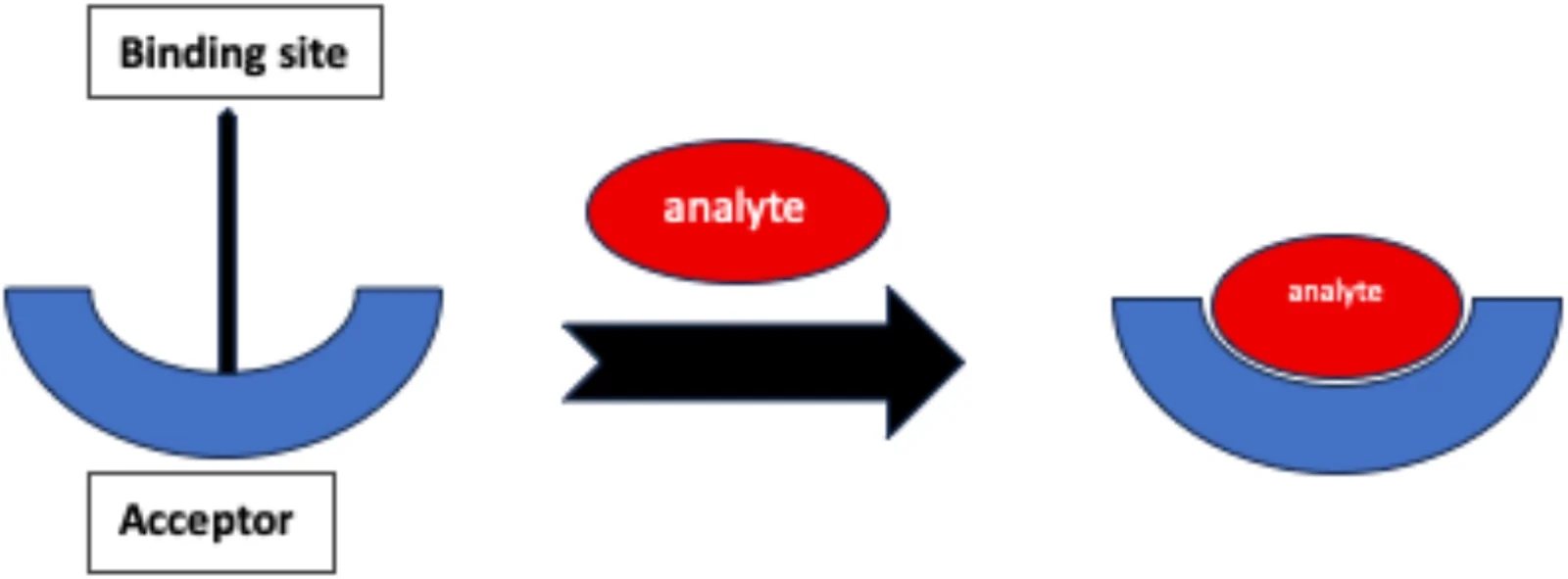
Colour transition observed upon addition of analyte to probe solution (left: probe alone; right: probe–analyte complex).

**Fig. 2:**
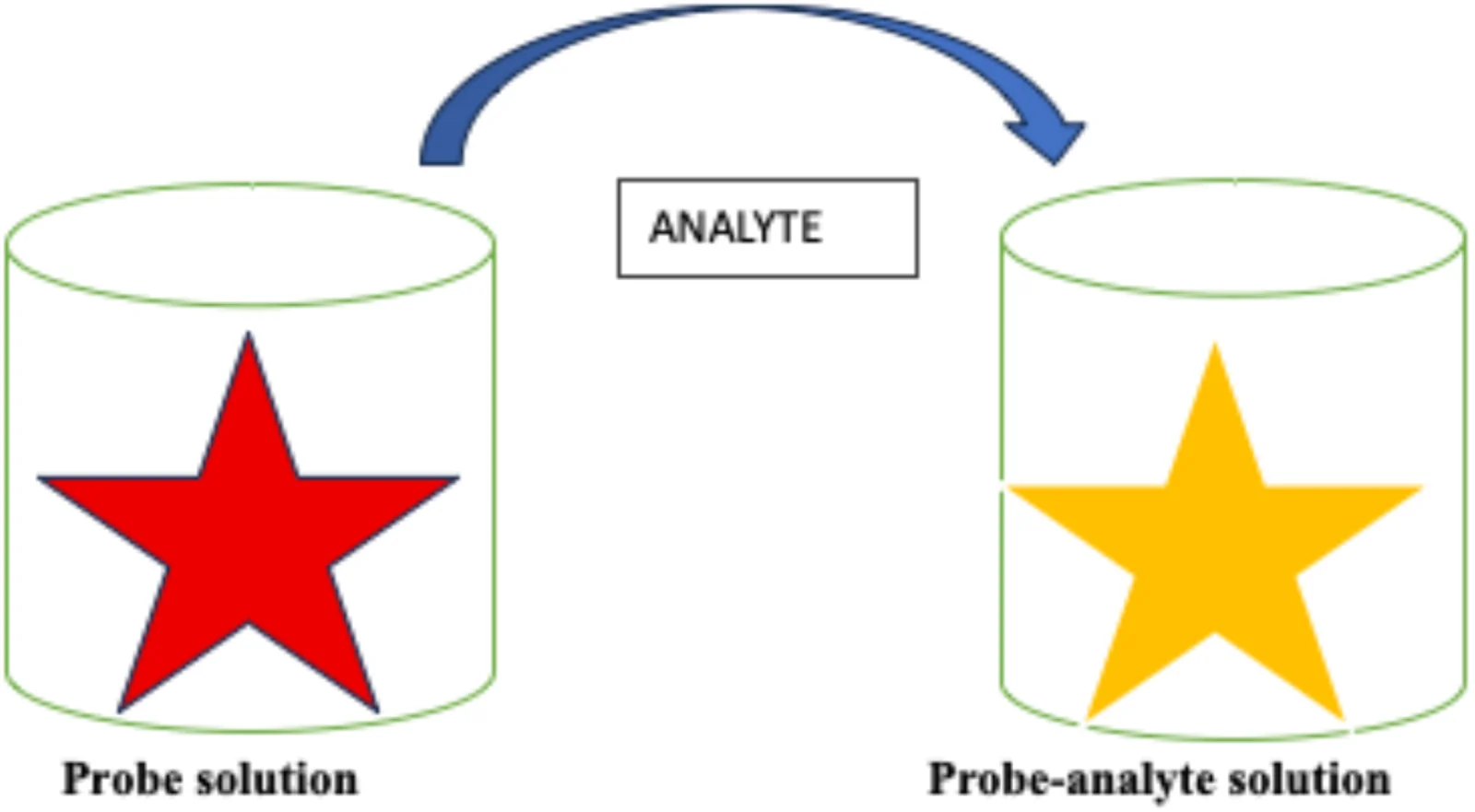
Schematic representation of analyte recognition by a Schiff base chemosensor, showing binding of the target analyte to the acceptor unit at the active binding site (left: free probe; right: probe–analyte complex).

Among optical sensing strategies, colorimetric detection exploits a probe–analyte interaction that produces a visible colour change, enabling naked-eye identification of the target species without the need for sophisticated instrumentation.^9–11^ This approach is particularly valued in resource-limited or point-of-care settings, where instrument-free detection is a practical necessity. The molecular recognition event underlying colorimetric detection is illustrated in Fig. 1, which shows the binding of the target analyte to the acceptor unit at the active binding site of the Schiff base chemosensor. Fluorescence-based sensing further enhances analytical performance by offering superior sensitivity, with detection limits frequently reaching the nanomolar to picomolar range. It also enables spatially resolved imaging in living cells.^12–14^ The fluorescence response of a probe may involve enhancement (turn-on), quenching (turn-off), or a ratiometric shift in emission wavelength. These responses arise from well-defined photophysical processes modulated upon analyte binding at the acceptor unit of the chemosensor, as schematically represented in Fig. 2.^15^

The photophysical mechanisms operative in fluorescence chemosensors have been extensively characterised and their relevance to specific probe architectures is well understood.^16–18^ In the context of Schiff base probes specifically, photoinduced electron transfer (PET) and intramolecular charge transfer (ICT) are the most frequently invoked mechanisms.^19,20^ PET-based probes exploit the lone pair on the imine nitrogen as an electron donor. Upon metal coordination, this lone pair is sequestered, suppressing PET and restoring fluorescence in a turn-on manner.^21,22^ ICT-based Schiff base sensors, which incorporate donor–pi–acceptor frameworks across the azomethine bond. These systems exhibit analyte-dependent shifts in both absorption and emission maxima, enabling ratiometric quantification and minimising background interference.^23–25^ Chelation-enhanced fluorescence (CHEF) is likewise central to Schiff base sensing, where metal ion coordination rigidifies the molecular framework around the C=N bond, suppresses non-radiative decay through restricted rotation, and thereby enhances emission intensity.^26,27^ Excited-state intramolecular proton transfer (ESIPT) occurs in Schiff bases bearing *ortho*-hydroxyl or *ortho*-amino substituents adjacent to the imine group. This mechanism produces characteristically large Stokes shifts, making these probes particularly suitable for bioimaging applications that require spectral separation from background autofluorescence is required.^28,29^ Fluorescence resonance energy transfer (FRET) has been applied in dual-fluorophore Schiff base systems for proximity-based sensing. Aggregation-induced emission enhancement (AIEE), which arises from restriction of intramolecular rotation (RIR) upon aggregation, has been exploited to develop Schiff base probes that remain analytically active in the solid state and in aqueous suspension. This approach overcomes the aggregation-caused quenching (ACQ) commonly observed in conventional organic fluorophores.^28–32^

Schiff bases, defined by the presence of the azomethine (–C=N–) linkage formed through condensation of a primary amine with an aldehyde or ketone, have occupied a prominent position in coordination chemistry since their introduction by Hugo Schiff in 1864.^33–36^ Their synthetic accessibility, structural tunability, and strong affinity for transition and heavy metal ions through the imine nitrogen and adjacent heteroatom donor sites have made them among the most widely explored ligand scaffolds in chemosensor design. Several reviews have addressed Schiff base sensors for specific analyte classes, including comprehensive treatments of metal ion detection,^37,38^ anion recognition,^39^ and fluorescent probes for biological thiols.^40,41^

More broadly, fluorescent chemosensors have been extensively reviewed from mechanistic, materials, and biomedical perspectives. Wu *et al.* summarised advances in molecular recognition, photophysical transduction, reaction-based sensing, and multifunctional probe design for analytical and biological applications.^42^ The emergence of aggregation-induced emission (AIEE) has further expanded optical sensing by overcoming aggregation-caused quenching and enabling efficient solid-state and aqueous sensing platforms.^43^ Recent reviews have also highlighted significant progress in fluorescent probes for reactive oxygen species and intracellular biochemical transformations, emphasising rational molecular design for enhanced selectivity, sensitivity, and bioimaging applications.^44,45^ Despite these advances, existing reviews primarily focus on specific sensing mechanisms, fluorophore platforms, or biological applications rather than the unique chemistry of Schiff base-derived probes. Consequently, a comprehensive review that critically compares Schiff base-based fluorescent and colorimetric chemosensors across both ionic and neutral analyte classes, integrating molecular design, sensing mechanisms, analytical performance, and practical applicability, remains lacking.

However, these accounts are largely restricted to individual analyte categories, specific fluorophore platforms, or narrow timeframes. None provides a unified treatment of the full mechanistic diversity of Schiff base sensing alongside a systematic comparison of performance metrics across analyte classes. The present review addresses this gap by offering a consolidated, critical analysis of Schiff base-derived colorimetric and fluorescent chemosensors across multiple analyte categories, integrating mechanistic interpretation with a structured evaluation of selectivity, sensitivity, and practical applicability.

While early Schiff base sensor research was dominated by transition and heavy metal ion detection, a scientifically significant and analytically challenging extension of this chemistry toward neutral and anionic analytes has gained considerable momentum over the past decade.^46,47^ The detection of neutral species, including biologically active small molecules such as amino acids, biothiols (cysteine, homocysteine, glutathione), vitamins, and reactive oxygen and nitrogen species, poses fundamentally distinct challenges compared to cationic analyte sensing. Unlike metal ions, neutral analytes do not directly coordinate to the imine nitrogen. Their recognition therefore requires alternative design strategies, such as chemodosimetric reactions, hydrogen-bond-directed interactions, or the incorporation of auxiliary recognition motifs, including boronic acid, urea, and amide groups, into the Schiff base scaffold.^48–51^ These analytes are also of considerable clinical and environmental importance. Biothiols regulate cellular redox homeostasis, and their dysregulation has been linked to neurodegenerative diseases, cardiovascular disorders, and cancer;^52,53^ reactive oxygen species are central mediators of oxidative stress; and vitamins such as B12 and thiamine are essential micronutrients whose deficiency carries serious neurological consequences.^54^ The expansion of Schiff base sensor design from ionic to neutral analyte detection therefore represents not merely a chemical curiosity but a practically motivated research frontier with direct implications for point-of-care diagnostics and environmental surveillance.

The photophysical responses of Schiff base chemosensors arise from one or more of the following well-defined mechanisms. In photoinduced electron transfer (PET)-based probes, an electron donor unit transfers an electron to the excited fluorophore in the absence of the analyte, suppressing emission; analyte binding blocks this transfer, restoring fluorescence in a turn-on response. Intramolecular charge transfer (ICT) probes incorporate electron-donor and electron-acceptor units connected through the azomethine bridge; analyte binding modulates the push–pull electronic balance, producing spectral shifts in absorption and emission. Chelation-enhanced fluorescence (CHEF) arises when metal ion coordination rigidifies the Schiff base scaffold by restricting rotation around the C=N bond, enhancing emission intensity. Excited-state intramolecular proton transfer (ESIPT) operates through an *ortho*-hydroxyl group positioned adjacent to the imine nitrogen; upon excitation, proton transfer from the enol to the keto tautomer generates a large Stokes shift that reduces background interference and enables ratiometric sensing. Förster resonance energy transfer (FRET) involves non-radiative energy transfer from an excited donor fluorophore to an acceptor, with efficiency governed by the donor–acceptor distance and spectral overlap; analyte binding modulates this transfer, producing ratiometric responses. Aggregation-induced emission enhancement (AIEE) exploits the restriction of intramolecular rotation (RIR) upon probe aggregation in aqueous media; probes that are weakly emissive in solution become strongly fluorescent in the aggregated state, enabling sensing in high water-fraction environments. The p*K*_a_ shift mechanism involves analyte-induced modification of the electron density at the imine nitrogen, shifting its basicity and switching the probe between non-emissive deprotonated and emissive protonated forms at physiological pH. Finally, direct imine bond cleavage operates through a chemodosimetric mechanism in which nucleophilic analytes cleave the C=N bond irreversibly, releasing a fluorescent fragment and providing reaction-based chemical selectivity. In terms of optical signal output, probes may exhibit fluorescence turn-on (emission enhancement upon analyte binding), turn-off (emission quenching), or ratiometric responses (simultaneous intensity increase at one wavelength and decrease at another), the last of which offers the advantage of self-calibration and reduced susceptibility to environmental interference.^55,56^

Despite the growing volume of literature, a critical and unified review that simultaneously addresses the mechanistic underpinnings of Schiff base sensing, the structural design principles governing selectivity, and the performance benchmarks across both ionic and neutral analyte categories is presently lacking. The present review addresses this gap by offering a consolidated, critical analysis of Schiff base-derived optical chemosensors, encompassing colorimetric, fluorescent, and optically transduced sensing platforms for neutral analytes. Unlike previous reviews, this article explicitly relates each probe class to Schiff base photophysics and compares sensing performance across different analyte classes. It also critically evaluates current limitations, including aqueous stability, selectivity in biologically complex media, and translational readiness. The subsequent sections cover the design principles and synthetic strategies for Schiff base chemosensors, the photophysical mechanisms operative in these systems, sensing applications toward neutral analytes including biologically relevant amino acids, proteins, vitamins, reactive small molecules, nitroaromatic compounds, neutral organic molecules, and neutral inorganic species, and current challenges and future perspectives for the field. The literature surveyed in this review was identified through structured searches of Scopus, Web of Science, PubMed, and Google Scholar, covering peer-reviewed journal articles and review articles published between January 2000 and March 2025, with particular emphasis on developments reported from 2010 onwards. Search terms included “Schiff base,” “chemosensor,” “fluorescent probe,” “colorimetric sensor,” “azomethine,” “neutral analytes,” and “bioimaging,” applied individually and in Boolean combinations. From an initial pool of over 1200 records, 130 studies were selected for inclusion based on relevance, data quality, and direct pertinence to Schiff base-derived optical sensing systems. Table 1 provides a consolidated mapping of each operative photophysical mechanism to the corresponding analyte classes, achievable detection limits, selectivity basis, and aqueous compatibility, serving as a unified mechanistic reference for the sensing systems discussed in the subsequent sections.^37,56^

**Table 1:** Mapping of photophysical mechanisms to analyte classes, representative probes, and key analytical performance parameters

Mechanism	Analyte class	LOD range	Detection strategy	Selectivity basis	Aqueous compatibility
PET	Biothiols, hydrazine, amino acids	nM–µM	Fluorescence	Metal displacement; imine lone-pair suppression upon analyte binding	Moderate (mixed organic–aqueous)
ICT	Hydrazine, amino acids	nM–µM	Dual-mode (colorimetric + fluorescence)	Donor–acceptor electronic balance shift across azomethine bridge	Moderate (DMF–H_2_O)
CHEF	Histidine, nitrophenols	µM	Fluorescence	Metal coordination-induced scaffold rigidification; restricted C=N rotation	Moderate (mixed solvent)
ESIPT	Hydrazine, selenocysteine, bioimaging	nM–µM	Fluorescence	*ortho*-OH directed proton transfer; large Stokes shift reduces background	Good (aqueous-compatible)
FRET	H_2_O_2_, white-light systems	Sub-µM	Fluorescence	Donor–acceptor energy transfer; distance/orientation-dependent	Ionic liquid/mixed solvent
AIEE	GSH, hydrazine, picric acid, NACs	ppb–µM	Dual-mode (colorimetric + fluorescence)	RIR upon aggregation; solid-state and aqueous suspension active	Good–excellent
p*K*_a_ shift	Sec, thiophenol, VDPs, HCl	nM–µM	Dual-mode (colorimetric + fluorescence)	Intrinsic nucleophilicity/acidity differences; electronic switching of emissive form	Good (aqueous-compatible)
Imine cleavage	GSH, mesotrione, formaldehyde	nM–µM	Fluorescence	Chemodosimetric C=N bond cleavage; reaction-based chemical selectivity	Variable (mixed to aqueous)

## 2 Sensing applications of Schiff base-based fluorescent and colorimetric sensors

Schiff base compounds occupy a distinctive position among optical chemosensor scaffolds because of their synthetic accessibility, photophysical tunability, and analyte-responsive electronic behaviour.^57–60^ The azomethine (–C=N–) functional group serves as a metal coordination site, a hydrogen-bond acceptor, and an electronic bridge between donor and acceptor fragments. These features enable efficient transduction of analyte–receptor interactions into measurable fluorescence or colour signals.^58,59^ This section surveys Schiff base-derived colorimetric and fluorescent chemosensors organised by analyte category, beginning with biologically relevant amino acids, proteins, and vitamins, which collectively represent the most extensively studied application domain. For each category, probes are discussed and compared critically on the basis of sensing mechanism, detection limit, solvent compatibility, and demonstrated real-sample applicability. A consolidated critical assessment and future perspectives are provided at the end of the section.

The analytical effectiveness of Schiff bases as sensing scaffolds arises from several convergent structural attributes. The reactive imine group functions both as a binding site and as an electronic conduit. It enables charge transfer and, for nucleophilic analytes, reaction-based chemodosimetric detection.^57,58,60^ The imine nitrogen is often reinforced by adjacent donor groups such as –OH, –NH_2_, or –S–. Together, these groups create a chelation cavity with tunable geometry and donor strength, making Schiff bases highly effective for metal ion complexation and displacement-based sensing strategies.^59,61^ The modular nature of Schiff base synthesis, achieved through condensation of a primary amine with an aldehyde or ketone under mild conditions, permits systematic substituent variation to optimise selectivity, detection range, and aqueous compatibility without fundamentally altering the sensing scaffold.^58,60^ Extended aromatic conjugation across the C=N bond promotes optical activity across the UV-visible spectrum and enables fluorescence responses that are sensitive to changes in molecular geometry, polarity, and coordination state upon analyte binding.^61^ Collectively, these features endow Schiff bases with access to multiple photophysical signalling pathways within a single molecular framework, including PET, ICT, ESIPT, CHEF, FRET, and AIEE. This versatility distinguishes them from that most other organic sensing scaffolds.^31,55,62,63^

### 2.1 Detection of amino acids, proteins, and vitamins

Arginine plays an indispensable role in a wide spectrum of biological processes including wound healing, immune function, and cell division, and its accurate quantification is therefore of considerable physiological importance.^64,65^ Two structurally distinct Schiff base systems have been reported for arginine sensing, and their comparison is instructive in illustrating the design trade-offs between simplicity and analytical performance.

**Fig. 3:**
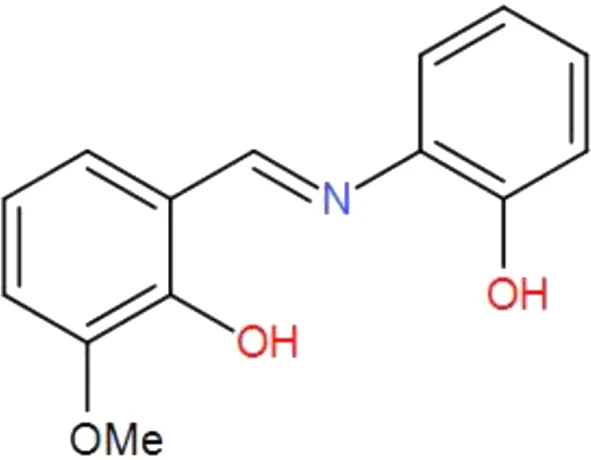
Structure of Schiff base probe S1 for arginine detection. The methoxysalicylaldehyde imine moiety acts as a hydrogen-bond donor/acceptor toward the guanidinium group of arginine, producing a new emission band at 450 nm with a confirmed 1 : 1 binding stoichiometry at the isosbestic point of 385 nm.

**Fig. 4:**
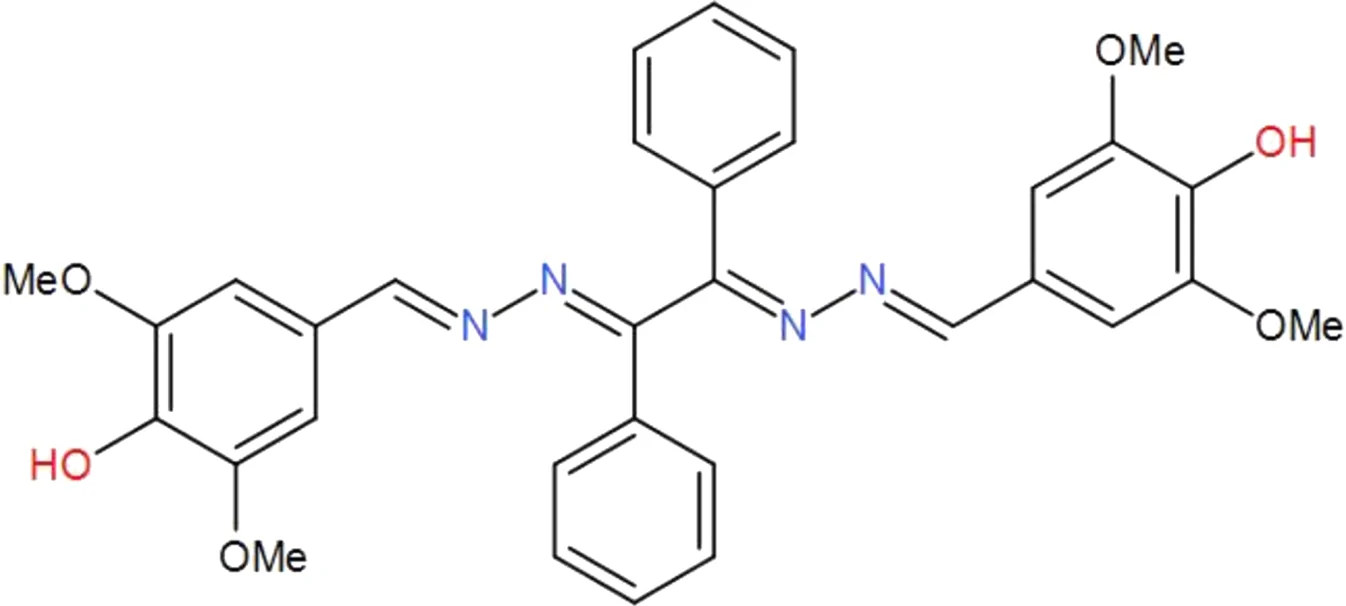
Structure of dual-mode Schiff base probe S2 for arginine detection. The free probe operates colorimetrically (LOD 0.67 µM), while its Pb^2+^ ensemble exploits metal-displacement-induced fluorescence quenching to achieve a 6700-fold improvement in sensitivity (LOD 0.1 nM).

Shang *et al.* developed probe S1, a methoxysalicylaldehyde-derived Schiff base that operates in DMSO–water medium. The probe detects arginine through hydrogen-bonding interactions with the guanidinium group.^66^ As shown in Fig. 3, the methoxysalicylaldehyde imine moiety provides both a hydrogen-bond donor through the phenolic –OH and a hydrogen-bond acceptor through the imine nitrogen. Together, these groups form a complementary recognition pocket for the guanidinium group of arginine. Incremental arginine addition produced an isosbestic point at 385 nm and enhanced emission at 450 nm, with Job's plot analysis confirming 1 : 1 host–guest stoichiometry. The operative concentration range of 0–200 µg mL^−1^ is compatible with physiological arginine levels (∼50–150 µM), and the mechanistic validation through theoretical calculations adds credibility to the proposed interaction model. However, the exclusive reliance on a mixed organic solvent system constrains its direct application in aqueous biological matrices. A markedly different approach was adopted by Ghorai *et al.*, whose Schiff base probe S2, shown in Fig. 4, forms a Pb^2+^ ensemble that provides a dual-mode colorimetric and fluorometric platform for arginine detection.^67^ S2 alone detects arginine colorimetrically through a colour change from colourless to yellow accompanied by a new absorption band at 430 nm, while the Pb^2+^–S2 complex affords fluorometric detection *via* quenching with a dramatically improved LOD of 0.1 nM compared to 0.67 µM for the free probe. This 6700-fold improvement in sensitivity through metal coordination exemplifies the power of the chelation-enhancement strategy. However, the use of Pb^2+^ as a sensing auxiliary introduces significant biocompatibility concerns that preclude clinical translation. In summary, S1 offers mechanistic clarity and moderate sensitivity, whereas S2 provides superior detection capability at the cost of biological suitability, highlighting a recurring trade-off in metal-assisted probe design.

**Fig. 5:**
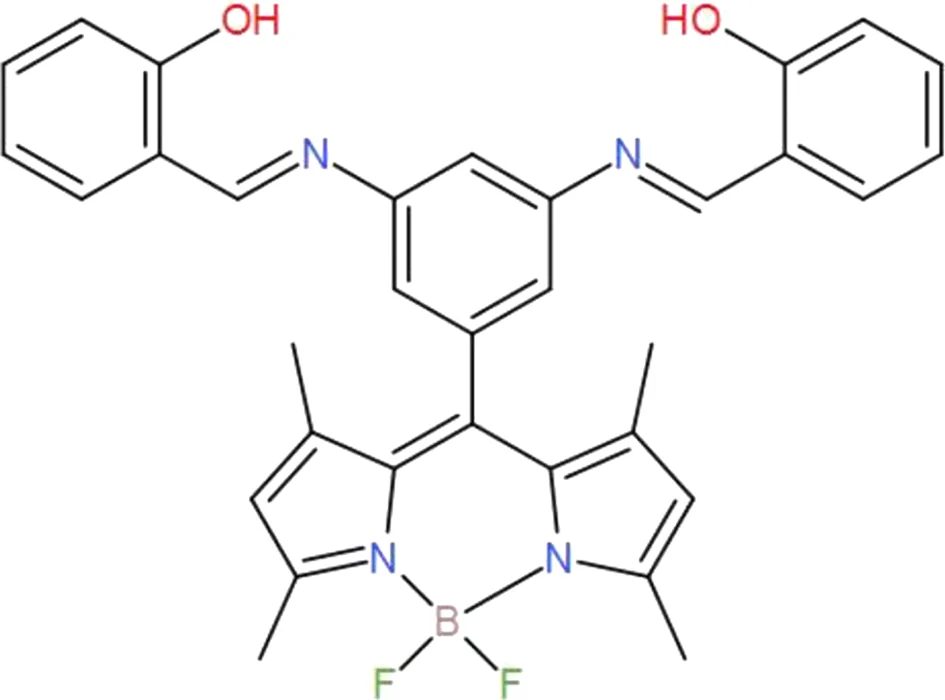
Structure of BODIPY–Schiff base probe S3 for cysteine and homocysteine detection. Upon Cu^2+^ coordination through the imine nitrogen, BODIPY emission is quenched *via* PET; selective thiol-mediated Cu^2+^ sequestration restores fluorescence (6-fold enhancement; LOD: Cys 0.17 µM, Hcy 0.25 µM), with characteristic UV-vis bands at 342 nm (charge transfer) and 499 nm (BODIPY π–π).

Cysteine and homocysteine are biologically critical aminothiols whose dysregulation is strongly linked to cardiovascular disease, neurodegeneration, and metabolic disorders.^52,53,68^ Li *et al.* reported a BODIPY–Cu^2+^ ensemble (S3) for the fluorescence detection of cysteine and homocysteine (Fig. 5). The probe operates through a competitive metal-displacement mechanism. The probe S3, whose structure is shown in Fig. 5, consists of a BODIPY fluorophore tethered to an imine-containing recognition unit. Upon addition of Cu^2+^, coordination through the imine nitrogen quenches BODIPY emission *via* photoinduced electron transfer, generating two characteristic UV-vis absorption bands at 342 nm (charge transfer within the imine coordination framework) and 499 nm (BODIPY π–π transition). Subsequent addition of cysteine or homocysteine sequesters Cu^2+^, restoring fluorescence selectively.^69^ The high affinity of cysteine and homocysteine for Cu^2+^ displaces the metal from the imine coordination site, suppressing PET and restoring fluorescence with a 6-fold enhancement. The LODs of 0.17 µM for cysteine and 0.25 µM for homocysteine fall comfortably below their respective intracellular concentrations (∼30–200 µM and ∼5–15 µM in plasma), confirming practical analytical utility. Notably, the system discriminates these thiols effectively from other amino acids, although the structural similarity between cysteine and homocysteine, reflected in the closely proximate LODs, suggests residual discrimination challenges that warrant further probe engineering. The dependence on Cu^2+^ and the mixed organic–aqueous medium remain the primary limitations.

**Fig. 6:**
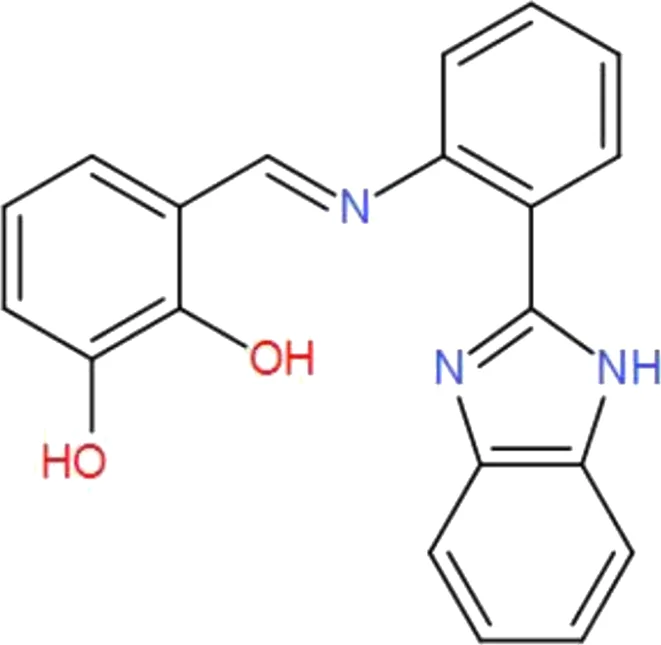
Structure of Schiff base probe S4 for histidine detection *via* competitive Cu^2+^ coordination. The preformed Cu^2+^–S4 complex acts as the active sensing unit; histidine binds competitively at the Cu^2+^ centre through its imidazole nitrogen and α-amino group (1 : 2 stoichiometry; *K*_a_ = 1.286 × 10^8^ M^−2^), quenching emission at 432 nm. Successfully validated in urine samples and live-cell imaging.

Histidine contributes to protein structure, enzyme catalysis, and metal ion homeostasis, and its selective sensing in biological fluids presents a genuine analytical challenge due to its structural similarity to other imidazole-containing species.^70,71^ Wang *et al.* constructed probe S4, whose structure is shown in Fig. 6, as the ligand component of a preformed Cu^2+^–Schiff base complex for histidine detection. Histidine binds competitively to the Cu^2+^ centre through its imidazole nitrogen and α-amino group in a multidentate manner, displacing the Schiff base ligand from the coordination sphere and inducing fluorescence quenching at 432 nm.^72^ The unusually high association constant of 1.286 × 10^8^ M^−2^ and a 1 : 2 (complex : His) binding ratio suggest strong, multidentate interaction of histidine with the Cu^2+^ centre through its imidazole nitrogen and α-amino group. A particularly valuable outcome of this work is the demonstrated applicability in urine samples and cell imaging, which establishes real-world relevance beyond typical proof-of-concept studies. The partial fluorescence quenching observed for glutamate and aspartate, however, indicates that the selectivity window narrows in the presence of competing acidic amino acids, a limitation that should be addressed before deployment in complex biological matrices.

**Fig. 7:**
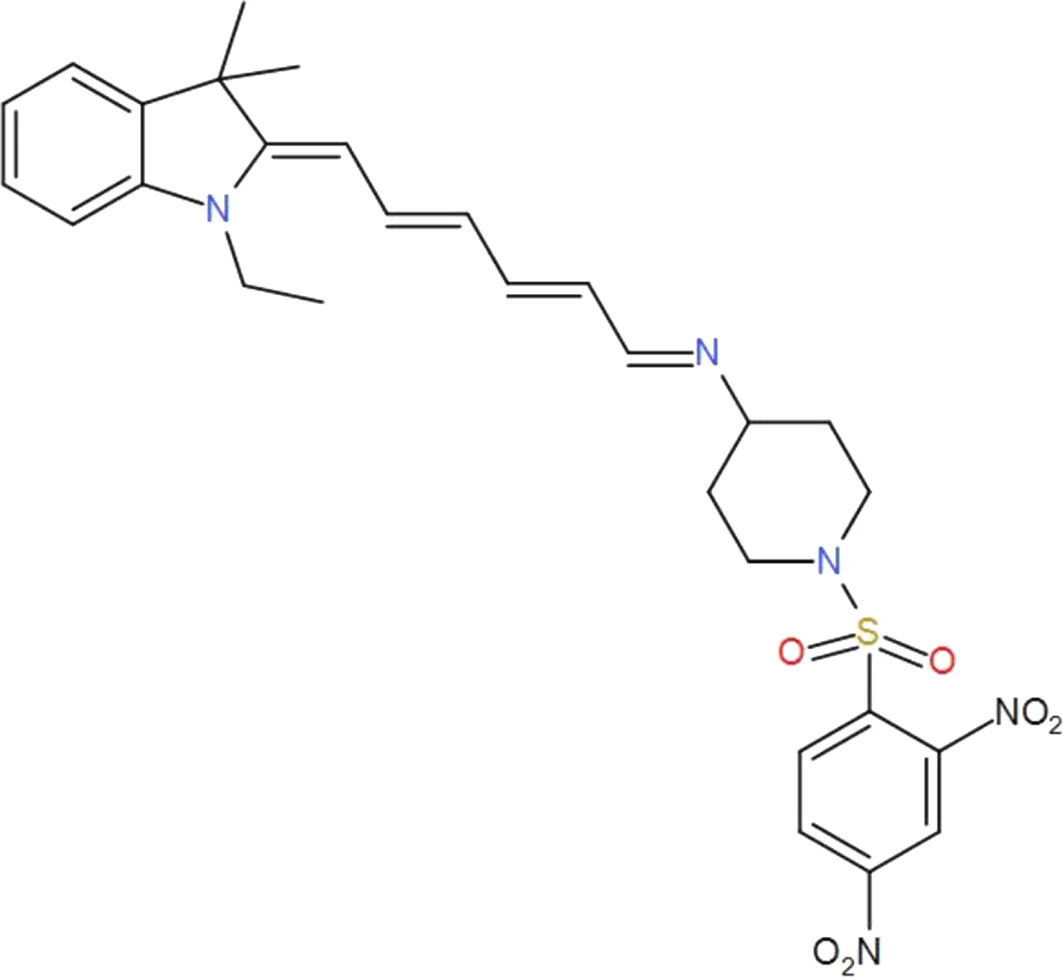
Structure of probe MC-Sec for selenocysteine (Sec) detection. The electron-withdrawing dinitrobenzenesulfonyl group on the imine nitrogen suppresses basicity, maintaining a p*K*_a_ of 6.40 and keeping the probe in its non-emissive deprotonated form at physiological pH 7.4. Sec-induced cleavage of this group raises the imine p*K*_a_ to 9.04, switching the probe to its emissive protonated form (21-fold fluorescence enhancement; LOD 68 nM; validated in live-cell imaging).

Selenocysteine (Sec) is the rarest of the canonical amino acids, present at low physiological concentrations and playing critical roles in antioxidant defence, thyroid hormone metabolism, and immune regulation.^73–77^ The challenge of selectively detecting Sec over the far more abundant structurally analogous cysteine is significant, as both species are nucleophilic aminothiols. The MC-Sec probe, whose structure is shown in Fig. 7, addresses this challenge through a p*K*_a_ shift mechanism that exploits the intrinsic nucleophilicity difference between selenium and sulfur. In its native state, the electron-withdrawing dinitrobenzenesulfonyl group on the imine nitrogen suppresses its basicity, maintaining a p*K*_a_ of 6.40 and keeping the probe in its non-emissive deprotonated form at physiological pH 7.4.^78^ Selective nucleophilic attack by the selenol group of Sec, which is considerably more reactive than the thiol group of cysteine under physiological conditions, cleaves the electron-withdrawing group from the imine. This reaction increases the p*K*_a_ from 6.40 to 9.04 and converts the imine nitrogen into its protonated, emissive form at physiological pH. This electronic switching mechanism generates a 21-fold fluorescence turn-on response. Because the response is based on intrinsic chemical reactivity rather than affinity or steric effects, the probe selectively detects Sec over cysteine and performs well in biologically complex environments. The LOD of 68 nM is well suited to the detection of Sec at physiological concentrations, and the successful confocal fluorescence imaging of both extracellular and intracellular selenol species in living cells demonstrates outstanding cellular permeability and probe stability within the intracellular environment.

Among the probes reviewed in this section, MC-Sec stands out for its mechanistically elegant, selectivity-by-reactivity approach, and it represents the most compelling demonstration of how intrinsic chemical reactivity differences between structurally similar analytes can be harnessed to achieve analytically robust discrimination without the need for metal coordination or macrocyclic receptor engineering.

**Fig. 8:**
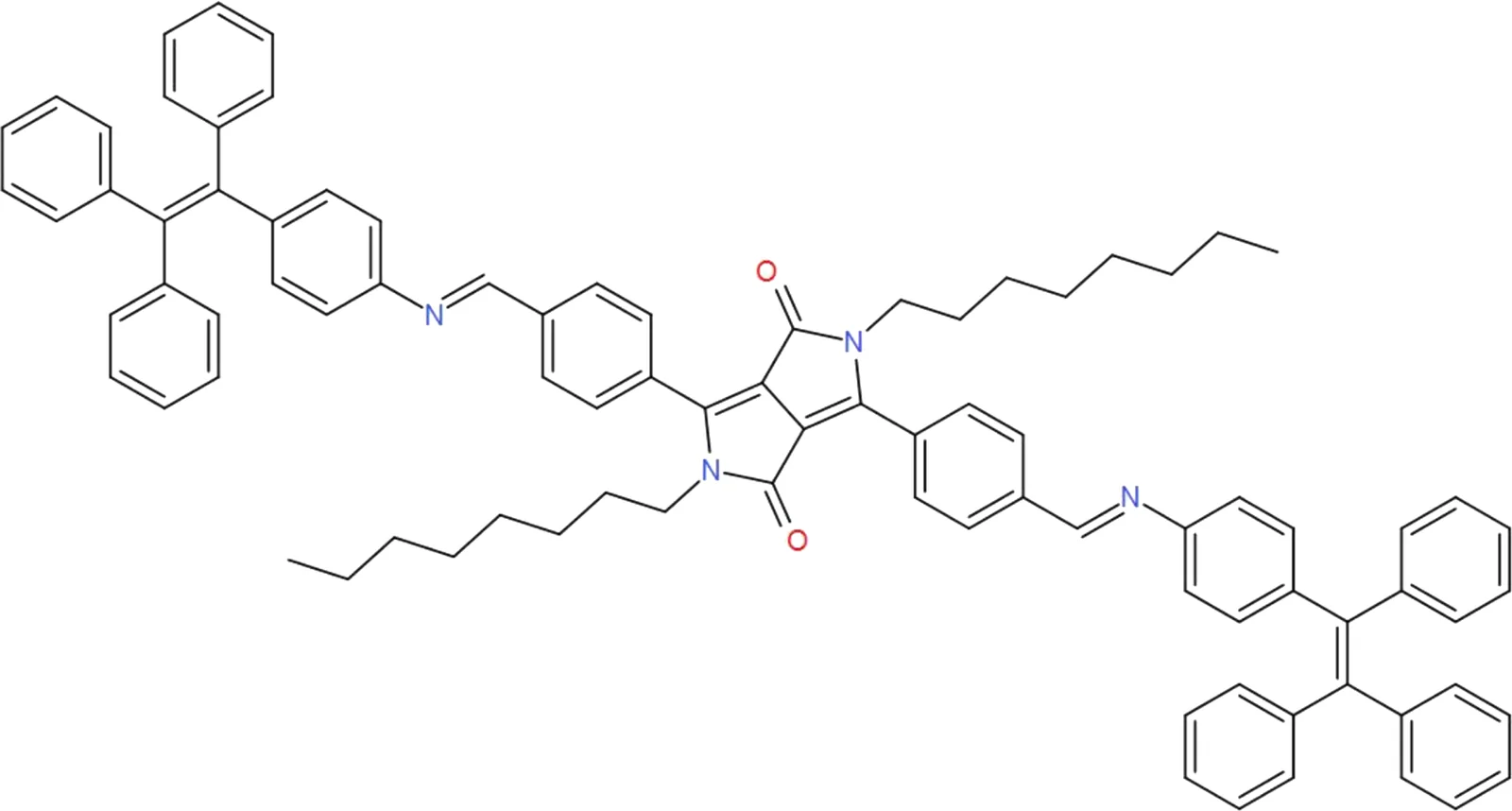
Structure of AIEE-active TPE-DPP Schiff base probe for glutathione (GSH) detection. The tetraphenylethylene (TPE) fragment provides AIEE behaviour through restriction of intramolecular rotation, while the diketopyrrolopyrrole (DPP) chromophore governs primary absorption and emission. GSH-induced disaggregation of probe nanoparticles (955 nm → 228 nm by DLS) activates a turn-on signal at 411 nm with a blue shift in absorption (590 → 578 nm); linear range 0–0.1 mM; LOD 0.05 µM; validated in live-cell imaging.

Glutathione (GSH), the most abundant intracellular non-protein thiol, functions as a primary antioxidant and redox buffer at millimolar concentrations, and its depletion is associated with neurodegeneration, liver damage, and immune dysfunction.^79–81^ Wang *et al.* reported a tetraphenylethylene-diketopyrrolopyrrole (TPE-DPP) Schiff base system, whose structure is shown in Fig. 8, that demonstrates the analytical advantage of aggregation-induced emission in aqueous environments.^82^ The TPE-DPP scaffold integrates two complementary functional units: the tetraphenylethylene (TPE) fragment provides AIEE behaviour through restriction of intramolecular rotation, while the diketopyrrolopyrrole (DPP) chromophore governs primary absorption and emission characteristics. In the aggregated state at high water fractions, restriction of intramolecular rotation of the TPE phenyl rings suppresses non-radiative decay and activates emission; the nanoparticle size, measured by dynamic light scattering, decreases progressively from 955 nm at 60% water fraction to 228 nm at 99% water fraction, reflecting a transition from loosely associated aggregates to compact nanoparticles. Upon addition of GSH, the thiol group reacts with the imine bond linking the TPE and DPP units. This reaction induces nanoparticle disaggregation, activates fluorescence at 411 nm, and produces a blue shift in absorption from 590 to 578 nm. Crucially, this design circumvents the aggregation-caused quenching that limits conventional BODIPY and fluorescein-based probes in aqueous suspension, making the TPE-DPP system inherently more suited to biological media than fluorophores reliant on molecular dispersion for emission. The LOD of 0.05 µM and linear range of 0–0.1 mM is analytically appropriate given the millimolar intracellular GSH abundance, and the application to live-cell imaging combined with the straightforward one-pot synthesis route makes this platform particularly attractive for further development toward point-of-care diagnostics. The main caveat is that performance is governed by aggregation dynamics and solvent composition, meaning that fluctuations in ionic strength, protein content, or pH within the cellular environment may alter nanoparticle assembly behaviour and introduce variability in quantitative measurements under genuine biological conditions.^82^

**Fig. 9:**
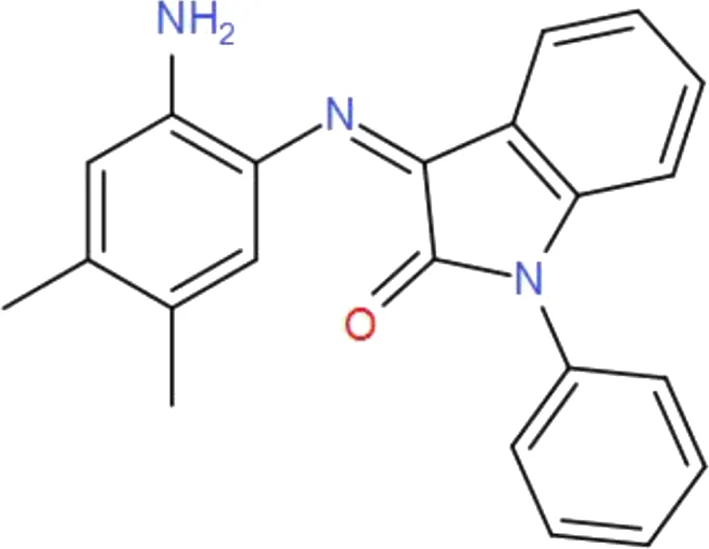
Isatin-functionalised Schiff base probe S5 for vitamin B12 detection *via* dynamic fluorescence quenching by the cobalamin cobalt centre; LOD 1.18 × 10^−9^ M, linear range 1–5 × 10^−7^ M; validated in blood and urine real samples.

Vitamin B12 (cobalamin) is essential for neurological function, DNA synthesis, and red blood cell formation, with deficiency manifesting as severe neuropathy and megaloblastic anaemia.^83–85^ Pradeep *et al.* synthesised an isatin-functionalised Schiff base probe (S5), whose structure is shown in Fig. 9, that operates through a dynamic fluorescence quenching mechanism in which the cobalt centre of cobalamin acts as the primary quenching species.^86^ The isatin moiety of S5 provides an extended conjugated framework that generates strong intrinsic fluorescence, while the Schiff base imine linkage connecting the isatin unit to the recognition arm provides the coordination geometry necessary for interaction with the cobalt centre of cobalamin. Notably, S5 was validated against genuine biological samples, specifically blood and urine, representing a meaningful and relatively rare step toward practical clinical utility in this category of probes, as most reported vitamin sensors are validated only in buffer or spiked aqueous solutions. Upon addition of vitamin B12, the Co^3+^ centre quenches the fluorescence of the isatin fluorophore. This occurs through a combination of the inner filter effect and heavy-atom-induced intersystem crossing, both of which accelerate non-radiative decay. This dual quenching pathway accounts for the exceptionally low LOD of 1.18 × 10^−9^ M achieved within a well-defined linear range of 1 × 10^−7^ to 5 × 10^−7^ M. The validation of S5 against genuine biological samples, specifically blood and urine, shown schematically in Fig. 9, represents a meaningful and relatively rare step toward practical clinical utility in this category of probes, as most reported vitamin sensors are validated only in buffer or spiked aqueous solutions. This level of sensitivity is significant because serum vitamin B12 concentrations (approximately 150–700 pM) fall within the clinically relevant detection range of the probe. Consequently, S5 ranks among the most analytically sensitive probes discussed in this section. A more thorough characterisation of selectivity against structurally related corrinoids and competing biomolecules present in blood matrices, including haemoglobin, transferrin, and other cobalt-containing species, would substantially strengthen the translational case for this probe.

**Scheme 1:**
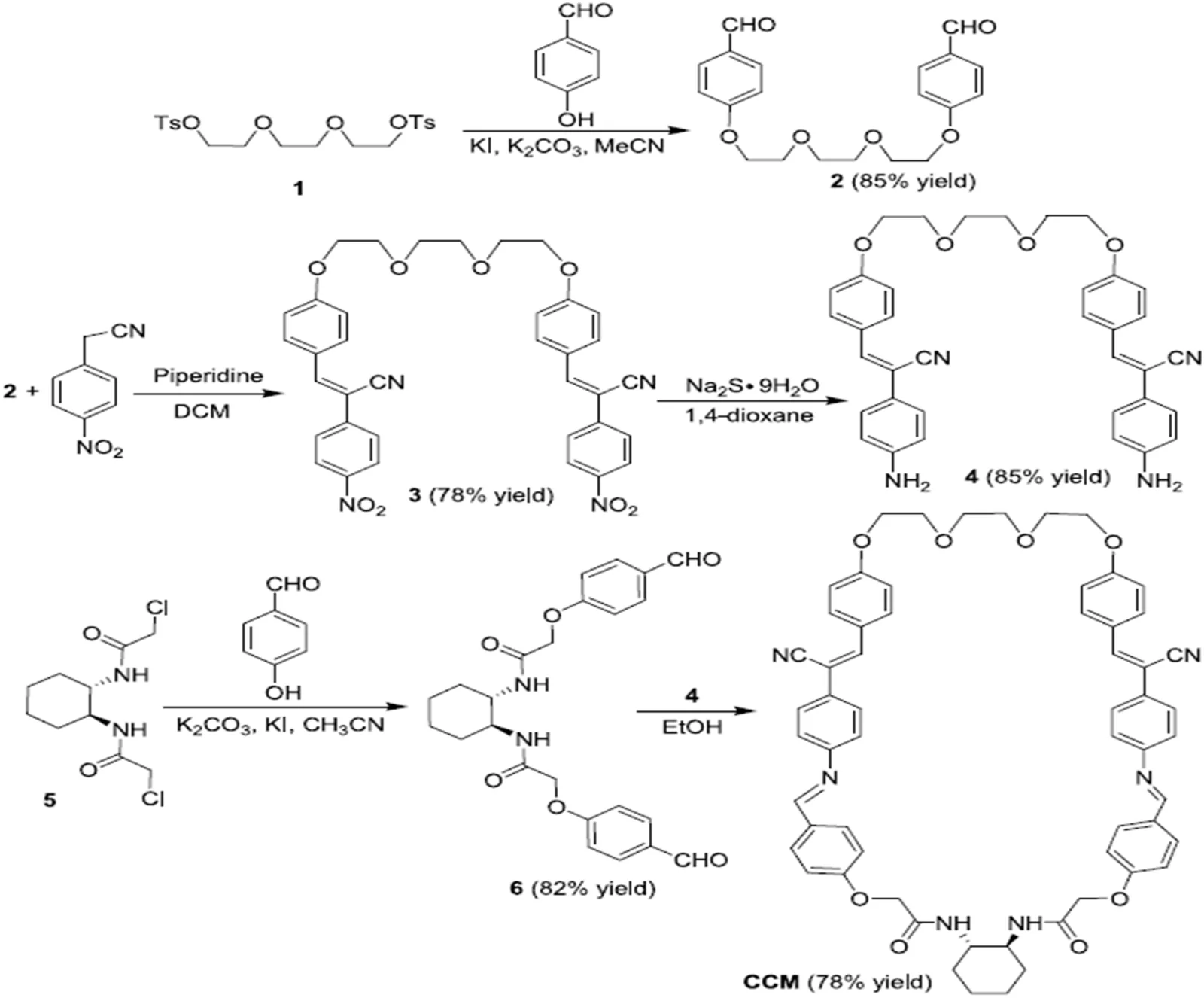
Multi-step synthesis of crown ether-based macrocyclic Schiff base S6 (CCM) for vitamin B1 (thiamine) detection, showing the convergent synthetic route from precursors 1–6 to the final macrocyclic product (overall yield 78%). The preorganised macrocyclic cavity formed is complementary to the thiazolium cation of thiamine, enabling host–guest binding that rigidifies the scaffold and activates green turn-on fluorescence (LOD 7.8 × 10^−7^ M); validated in peanuts and vitamin B1 tablets.^88^ Adapted/reproduced from ref. 88 with permission from Elsevier,^88^ Copyright 2022.

Vitamin B1 (thiamine) is required for carbohydrate metabolism and neurological function; its deficiency causes Wernicke's encephalopathy, memory loss, and gastrointestinal disturbance.^54,87^ In a mechanistically distinct approach, Chen *et al.* synthesised a crown ether-based macrocyclic Schiff base (S6) *via* a convergent multi-step strategy, as shown in Scheme 1.^88^ The synthesis proceeds through sequential functionalisation steps, assembling the macrocyclic framework through condensation of a dialdehyde-functionalised crown ether precursor with a diamine component, incorporating imine bonds directly into the ring structure. This creates a preorganised binding cavity whose size, geometry, and donor atom arrangement are complementary to the thiazolium cation of thiamine, enabling host–guest recognition and turn-on fluorescence response. This synthetic design is mechanistically significant because the macrocyclic preorganisation reduces the conformational entropy penalty upon guest binding relative to open-chain receptors, thereby enhancing binding affinity and selectivity without requiring metal coordination. Upon thiamine binding, the thiazolium cation of the guest is accommodated within the crown ether cavity through a combination of electrostatic interactions between the positively charged thiazolium ring and the polyether oxygen donors, and CH–π or π–π interactions between the aromatic thiazolium and the imine-containing walls of the macrocycle, as illustrated in the right panel of Scheme 1. This binding event rigidifies the macrocyclic framework, suppresses conformational non-radiative decay through restricted intramolecular motion of the imine-containing walls, and activates a green fluorescence turn-on response with a LOD of 7.8 × 10^−7^ M.

The receptor design philosophy here, employing host–guest complementarity rather than metal coordination, is notably different from the majority of probes reviewed in this section and represents a more biomimetic approach to analyte recognition that avoids the biocompatibility concerns associated with heavy metal auxiliaries.

Importantly, the successful application of S6 in complex food matrices including peanuts and vitamin B1 tablets, validated by FTIR, mass spectrometry, and ^1^H NMR characterisation of the host–guest complex, demonstrates direct relevance to food quality control and nutritional analysis and confirms that the macrocyclic binding cavity retains its selectivity and sensitivity in the presence of the diverse chemical background of real food samples.

Human serum albumin (HSA) is a primary biomarker of renal function, with elevated urinary HSA (albuminuria) serving as an early diagnostic indicator of chronic kidney disease.^89,90^ Wang *et al.* reported a hydrazide Schiff base self-assembled nanoprobe (NPC: *N*-((7-(diethylamino)-2-oxo-2*H*-chromen-3-yl)methylene)pyrazine-2-carbohydrazide) that exhibits a fluorescence turn-on response upon migration into the hydrophobic binding pockets of HSA, exploiting the polarity-sensitive emission of the coumarin-pyrazine fluorophore.^91^ The LOD of 0.56 mg L^−1^ in urine samples is clinically meaningful, as the threshold for microalbuminuria is defined at 30 mg L^−1^, providing a sensitivity margin of approximately 50-fold. Validation through Job's plot analysis, fluorescence quenching studies, and HSA degradation experiments confirms specific probe–protein binding rather than non-specific environmental effects.

Vicinal dithiol-containing proteins (VDPs) are overexpressed in tumour cells and have been identified as potential cancer biomarkers, making their selective detection in cellular environments analytically and clinically significant.^92,93^ A red-emitting merocyanine-based Schiff base probe, MCAs, achieves selective VDP detection through a microenvironment-driven p*K*_a_ shift: in free solution, MCAs is weakly fluorescent; upon migration into the hydrophobic pockets of reduced bovine serum albumin (rBSA), the local polarity increase shifts the probe toward its protonated Schiff base form, generating a bathochromic absorption at 634 nm and enhanced emission at 657 nm.^94^ The red-emitting, turn-on character of this response is particularly advantageous for live-cell imaging, as it minimises overlap with cellular autofluorescence and improves depth penetration. Relative to the other probes in this section, MCAs is unusual in exploiting protein microenvironment effects rather than direct chemical reactivity, and the physiological compatibility of this approach supports its further development for tumour imaging applications.

Analysis of Table 2 reveals several non-obvious trends that extend beyond the individual probe descriptions. First, the most analytically compelling probes are those that exploit intrinsic differences in chemical reactivity rather than binding affinity alone. For example, MC-Sec selectively detects selenocysteine over cysteine by exploiting the higher nucleophilicity of selenium. Likewise, the Pb^2+^ complex of S2 achieves sub-nanomolar arginine detection through the preferred coordination geometry of lead with the guanidinium group. This reactivity-based approach represents a more robust strategy than affinity-based displacement, where cross-reactivity among structurally similar species is an intrinsic limitation. Second, an inverse relationship exists between aqueous compatibility and analytical sensitivity. The probes with the lowest detection limits, such as the S2–Pb^2+^ complex (0.1 nM) and S5 (1.18 × 10^−9^ M), operate in mixed or partially organic media. In contrast, more aqueous-compatible systems, including TPE-DPP, MCAs, and NPC, generally sacrifice sensitivity in favour of improved biological applicability.

**Table 2:** Comparative summary of Schiff base-based sensing systems for amino acids, proteins, and vitamins

Probe	Analyte	Detection strategy	Mechanism	LOD	Solvent system	Real sample validation	Key limitation	Reversibility/reusability
S1 (ref. 66)	Arginine	Fluorescence	H-bonding (1 : 1)	0.67 µM (free probe)	DMSO–H_2_O	Biochemical assays only	Mixed solvent; no real biological matrix	Reversible (non-covalent H-bonding)
S2/Pb^2+^–S2 (ref. 67)	Arginine	Dual-mode (colorimetric + fluorescence)	Metal-ion displacement; dual-mode	0.67 µM (colorimetric); 0.1 nM (fluorometric)	Aqueous-compatible	Analytical samples	Pb^2+^ toxicity; biocompatibility concern	Irreversible (metal-displacement; single-use)
S3 (ref. 69)	Cys/Hcy	Fluorescence	PET suppression *via* Cu^2+^ displacement	0.17 µM (Cys); 0.25 µM (Hcy)	Mixed organic–aqueous	Not demonstrated	Limited Cys/Hcy discrimination; organic solvent	Reversible (competitive Cu^2+^ displacement)
S4 (ref. 72)	Histidine	Fluorescence	Competitive coordination quenching (ICT)	*K*_a_ = 1.286 × 10^8^ M^−2^	Mixed solvent	Urine; cell imaging	Turn-off mode; partial interference from Glu/Asp	Reversible (competitive coordination)
MC-Sec^78^	Selenocysteine	Fluorescence	p*K*_a_ shift; nucleophilic addition	68 nM	Aqueous-compatible	Live-cell imaging (extracellular & intracellular)	Limited biological validation in complex matrices	Irreversible (nucleophilic addition; chemodosimeter)
TPE-DPP^82^	Glutathione	Fluorescence	AIEE-based disaggregation	0.05 µM	High water fraction (aqueous)	Live-cell imaging	Aggregation variability; solvent-dependent performance	Irreversible (imine cleavage by GSH)
S5 (ref. 86)	Vitamin B12	Fluorescence	Dynamic fluorescence quenching	1.18 × 10^−9^ M	Mixed solvent	Blood and urine	Selectivity *vs.* related corrinoids not fully characterised	Not demonstrated
S6 (ref. 88)	Vitamin B1	Fluorescence	Host–guest binding; turn-on	7.8 × 10^−7^ M	Organic–aqueous	Peanuts; vitamin B1 tablets	Biological fluid validation absent	Reversible (non-covalent host–guest)
NPC^91^	HSA	Fluorescence	Binding-induced turn-on; polarity-sensitive emission	0.56 mg L^−1^ (urine)	Aqueous/buffer	Human urine	Selectivity *vs.* other serum proteins requires further validation	Reversible (non-covalent protein binding)
MCAs^94^	VDPs	Fluorescence	Microenvironment-driven p*K*_a_ shift; red turn-on	Sub-µM protein conc.	Aqueous/physiological	Live-cell imaging	Specificity *vs.* other thiol-rich proteins needs broader validation	Reversible (non-covalent; p*K*_a_ shift)

This trade-off underscores that sensitivity optimisation and aqueous operability are currently competing rather than complementary design objectives in this field. Third, only a few probes demonstrated validated performance in real clinical or food samples. These include S4 (urine), S5 (blood and urine), S6 (food products), and NPC (human urine). All incorporate a secondary binding element, such as a coordinated metal ion, a macrocyclic cavity, or a self-assembled nanostructure, which enhances both selectivity and matrix tolerance. This pattern suggests that multi-component probe architectures are more likely to achieve real-world applicability than single-component systems, a design principle that should guide future molecular engineering efforts.

Across the probes surveyed in this section, several recurring analytical limitations are apparent. The most significant limitation is the widespread reliance on mixed organic–aqueous solvent systems. This reduces compatibility with biological fluids and limits point-of-care applications. This limitation is exacerbated by the known susceptibility of the imine bond to hydrolysis under acidic or strongly aqueous conditions, which can erode probe integrity during extended biological assays. A second systemic weakness is the insufficient discrimination among structurally similar analytes within the same chemical class: cysteine and homocysteine are rarely discriminated to clinical standards, and most amino acid probes show partial interference from competing species at physiologically relevant concentrations. Finally, although many studies report excellent detection limits, relatively few evaluate operational stability, long-term storage, or reproducibility in repeated real-sample analyses. These characteristics are essential for practical applications.

Looking forward, several design directions are most likely to yield meaningful analytical advances. Developing water-stable imine architectures through electron-withdrawing substituents, macrocyclisation, or imine-to-amide conversion could improve stability without compromising sensing performance. Ratiometric probe designs, in which the sensing signal is expressed as a ratio of two emission bands rather than a single intensity change, would improve quantitative accuracy in complex biological matrices by correcting for probe concentration variability and background interference. Integrating Schiff base sensing elements into paper strips, nanoparticles, solid-state devices, or smartphone-based sensing platforms would facilitate practical field applications. Finally, computational methods such as molecular docking and DFT calculations could be used to predict analyte–probe interactions before synthesis. This approach would accelerate the development of probes with improved selectivity. Progress on these fronts, combined with rigorous biological validation in physiologically complex matrices, will be necessary to advance Schiff base-based biomolecular sensing from proof-of-concept demonstrations to clinically and environmentally relevant analytical platforms.

This section has surveyed ten Schiff base-derived fluorescent and colorimetric sensing systems targeting amino acids (arginine, cysteine, homocysteine, histidine, selenocysteine, glutathione), vitamins (B12 and B1), and proteins (HSA and VDPs). Collectively, these studies demonstrate that Schiff base probes can achieve competitive detection limits for a diverse range of biologically important analytes. Their sensing mechanisms include metal displacement, p*K*_a_-shift, host–guest recognition, AIEE-based disaggregation, and microenvironment-sensitive fluorescence. Real-sample validation, achieved in blood, urine, food matrices, and living cells by selected probes, demonstrates the practical potential of this scaffold class. Despite these advances, challenges remain in aqueous operability, discrimination between structurally similar analytes, and comprehensive translational validation. Addressing these limitations should be a priority for future research. The following sections extend this analysis to Schiff base probes for reactive small molecules, environmental pollutants, and volatile organic compounds.

### 2.2 Thiophenol detection

Thiophenol (PhSH) and its derivatives are highly reactive aromatic thiols that are widely employed as industrial intermediates in the manufacture of pesticides, polymers, and pharmaceuticals. Despite their industrial utility, thiophenols are classified by the United States Environmental Protection Agency (USEPA) as priority environmental pollutants (waste code P014), with a median lethal concentration of 0.01–0.4 mM for fish and a lethal dose (LD_50_) of approximately 46 mg kg^−1^ in rodents.^95,96^ Chronic exposure in humans causes muscular weakness, central nervous system damage, hind limb paralysis, respiratory distress, and in severe cases, coma and death. A major analytical challenge is distinguishing thiophenol from structurally similar aliphatic thiols, such as cysteine and glutathione. This selectivity arises from the greater nucleophilicity of aromatic thiols under physiological conditions. This chemical distinction has been exploited in the design of reaction-based fluorescent probes that utilise nucleophilic aromatic substitution (SNAr) at activated electrophilic sites as the selectivity-conferring element, with 2,4-dinitrobenzenesulfonyl and sulfonamide groups emerging as the dominant recognition units in the literature.^96^

**Fig. 10:**
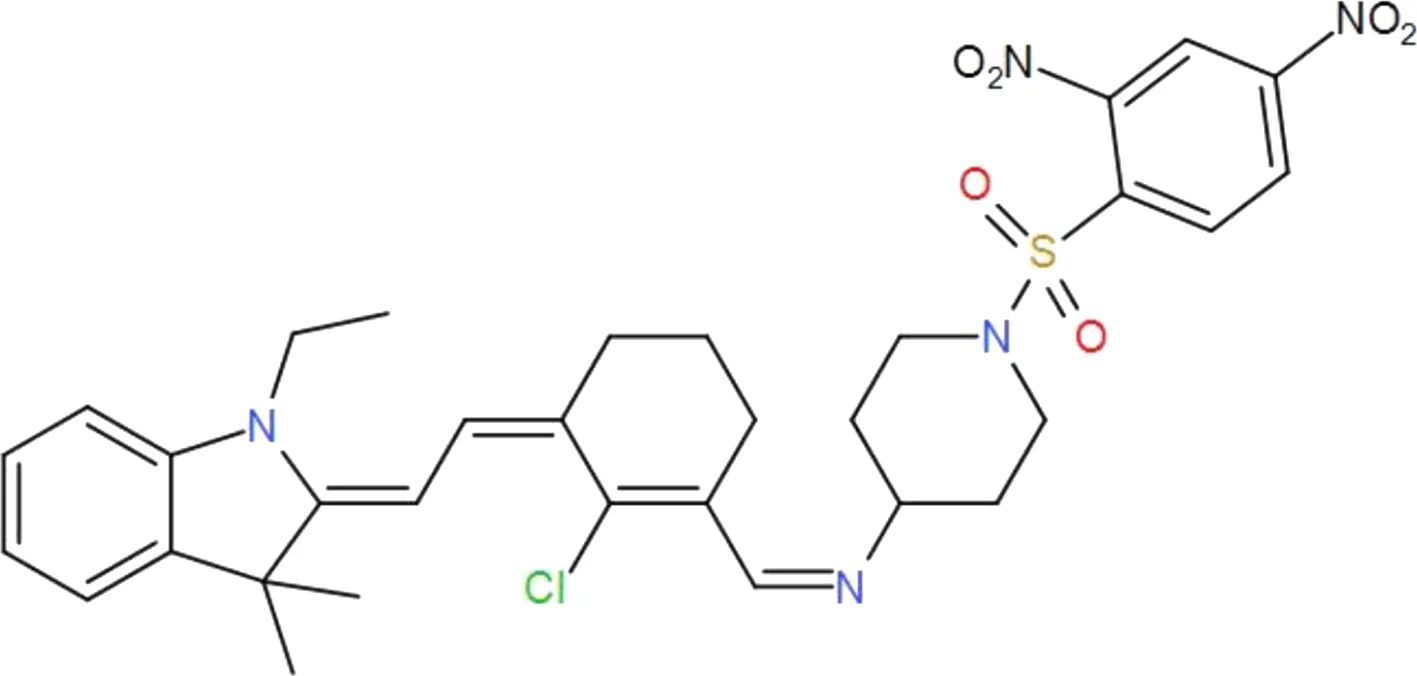
Merocyanine-based Schiff base probe SBMD for thiophenol detection *via* a p*K*_a_ shift mechanism. The DNBS recognition unit suppresses merocyanine emission in the native state by lowering the nitrogen p*K*_a_; nucleophilic aromatic substitution by thiophenol cleaves the sulfonamide, restoring the protonated emissive form at 627 nm (LOD 15 nM, linear range 0.2–3 µM); successfully applied to live-cell imaging with demonstrated selectivity over aliphatic thiols.

Within the Schiff base framework, Zhang *et al.* developed a merocyanine-based Schiff base probe (SBMD) for thiophenol detection in aqueous media and living cells *via* a p*K*_a_ shift mechanism.^97^ As depicted in Fig. 10, the SBMD probe incorporates a merocyanine fluorophore as the signalling unit and a 2,4-dinitrobenzenesulfonamide (DNBS) group as the electrophilic recognition unit. In its native state, the DNBS moiety withdraws electron density from the merocyanine nitrogen through the sulfonamide linkage, lowering its p*K*_a_ and maintaining the probe in its non-emissive deprotonated form at physiological pH. Thiophenol undergoes nucleophilic aromatic substitution at the DNBS unit, cleaving the sulfonamide linkage. This reaction restores the protonated emissive form of merocyanine and produces strong fluorescence at 627 nm. This mechanism confers intrinsic selectivity for aromatic thiols over aliphatic thiols, as the rate of SNAr is orders of magnitude faster for the more nucleophilic ArSH at neutral pH. The probe exhibited a linear response across 0.2–3 µM with a LOD of 15 nM, and was successfully applied to live-cell fluorescence imaging with clear intracellular signal, confirming cellular permeability and biocompatibility. The principal strength of SBMD lies in its chemically driven selectivity, which is based on intrinsic reactivity rather than binding affinity. However, the irreversible SNAr reaction prevents sensor reuse and may allow interference from other strong nucleophiles in complex samples. Compared with the broader landscape of thiophenol probes, many of which employ similar DNBS recognition units, SBMD is notable for integrating the merocyanine p*K*_a_ shift mechanism, which provides a sharper and more pH-stable turn-on response than simple DNBS cleavage-based fluorescence release.

Across the thiophenol sensing literature, the DNBS-based SNAr strategy dominates due to its predictable and rapid reactivity with aromatic thiols. However, the intrinsically irreversible nature of this reaction limits probe reusability and quantitative accuracy in dynamic sensing scenarios. A significant and under addressed challenge is discrimination from polysulfide species and H_2_S, which can also participate in SNAr reactions at activated electrophilic sites under certain conditions, compromising selectivity in complex environmental matrices such as wastewater. Future Schiff base probe design for thiophenol should explore reversible binding strategies, potentially through reversible coordination to soft Lewis acidic metal centres, and ratiometric detection modes that correct for background variability in environmental samples.

Hydrazine (N_2_H_4_) is a colourless, highly reactive liquid used industrially as a rocket propellant, boiler water treatment agent, polymer foaming agent, and agricultural chemical intermediate. It is classified by the U.S. Environmental Protection Agency as a Group B2 probable human carcinogen and by the International Agency for Research on Cancer (IARC) as Group 2A (probably carcinogenic to humans), based on evidence of lung, liver, and nasal tumour induction in multiple rodent species.^98^

Acute human exposure causes irritation of the eyes and respiratory tract, temporary blindness, dizziness, and neurological symptoms, while chronic exposure is associated with hepatotoxicity, genotoxicity, and carcinogenesis. The U.S. EPA has set an inhalation unit risk estimate of 4.9 × 10^−4^ (µg m^−3^)^−1^, corresponding to an effective environmental threshold of approximately 10 ppb (∼300 nM) in water, making nanomolar detection sensitivity analytically necessary rather than merely desirable.^98^ Reviews by Wang *et al.*^99^ and Zhang *et al.*^100^ have comprehensively documented the general landscape of hydrazine fluorescent sensors; the present section focuses specifically on Schiff base-derived systems, which exploit the unique reactivity of the azomethine group toward hydrazine through condensation, cleavage, and electron transfer modulation. The probes surveyed here are organised by mechanism type, from simpler PET-inhibition and Schiff base cleavage systems through to ICT-modulated and AIEE-based architectures, to provide a coherent mechanistic narrative of the field's development.

**Fig. 11:**
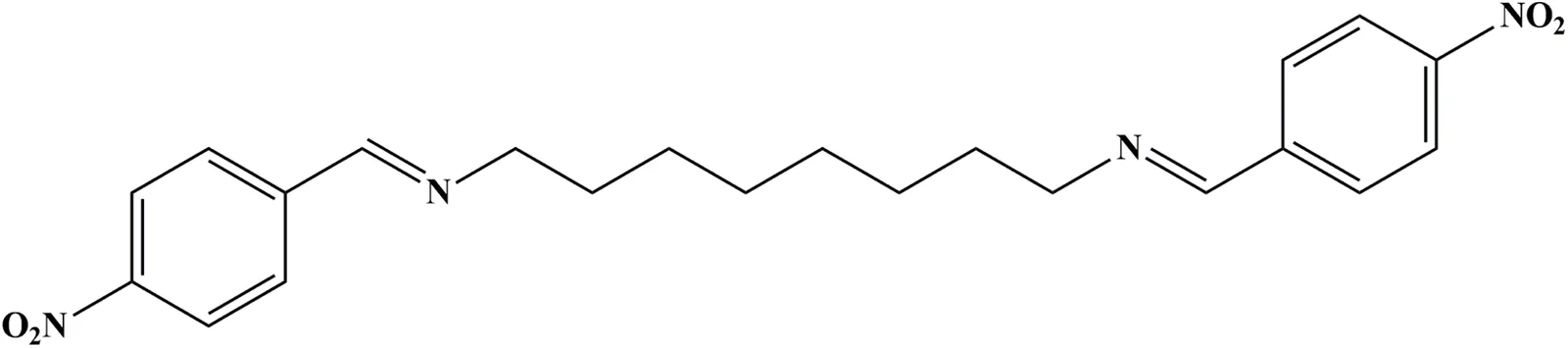
Benzaldehyde-based Schiff base chemosensor SBB for hydrazine detection. Hydrazine binds *via* hydrogen bonding at the imine nitrogen in a 1 : 2 stoichiometry (*K*_a_ = 6.67 × 10^4^ M^−1^, LOD 9.77 × 10^−8^ M), producing a 10.5-fold fluorescence turn-off and a colourless-to-yellow colour change across pH 4–12; two isosbestic points confirm clean species conversion; applied to water samples.

The simplest mechanistic strategy for hydrazine detection with Schiff base probes exploits direct condensation between hydrazine and the aldehyde component of the probe or reaction at the imine bond itself. Mondal *et al.* developed a benzaldehyde-based Schiff base chemosensor (SBB) that detects hydrazine through a 10.5-fold fluorescence turn-off response accompanied by a visible colour change from colourless to yellow, observable across a broad pH window of 4–12.^101^ UV-vis titration of SBB with hydrazine produced two well-defined isosbestic points, indicating clean conversion of a single reactant into a single product without detectable intermediates. This behaviour supports the proposed sensing mechanism and enables quantitative application of the probe. The structure of SBB is shown in Fig. 11. Hydrazine binds SBB through hydrogen-bonding interactions with the imine nitrogen in a 1 : 2 (probe : hydrazine) stoichiometry, confirmed by Job's plot, with an association constant of 6.67 × 10^4^ M^−1^ and a LOD of 9.77 × 10^−8^ M. The mechanism was thoroughly validated by DFT, NMR, and HRMS studies. Real water sample applicability was demonstrated, confirming practical environmental monitoring potential. The turn-off fluorescence mode, however, is an inherent analytical limitation, as decreasing signal is less reliable in complex matrices where background fluorescence can obscure quenching responses.

**Fig. 12:**
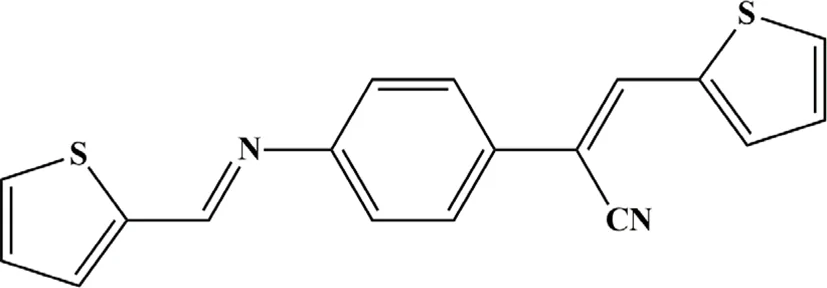
Thiophene-cyanodistyrene Schiff base TBA for hydrazine ion detection *via* PET inhibition. Hydrazine binding in 2 : 1 stoichiometry at the imine nitrogen suppresses PET from the thiophene donor, activating turn-on fluorescence (*K*_a_ = 5.25 × 10^7^ M^−1^, LOD 1.05 × 10^−7^ M); mechanism validated by DFT, NMR, FTIR, and HRMS; applied to tap water and Minjiang River samples.

A complementary PET-based approach was adopted by Huang *et al.*, who developed a thiophene-cyanodistyrene Schiff base (TBA) for hydrazine ion detection in aqueous solution *via* a turn-on mechanism.^102^ In the free probe, strong PET from the thiophene donor to the acceptor cyanostyrene unit quenches fluorescence; hydrazine binding in a 2 : 1 stoichiometry at the imine coordination sites suppresses this PET process, restoring emission with a binding constant of 5.25 × 10^7^ M^−1^ and a LOD of 1.05 × 10^−7^ M. The sensing mechanism of TBA, whose structure is shown in Fig. 12, was independently confirmed by DFT calculations, NMR, FTIR, and HRMS, providing multi-technique mechanistic validation that strengthens confidence in the proposed binding mode. Critically, the probe was applied to the detection of hydrazine in tap water and Minjiang River samples, making TBA one of the few Schiff base hydrazine probes with demonstrated performance in genuine environmental field samples rather than spiked laboratory solutions. Comparing SBB and TBA, the turn-on character of TBA's PET-inhibition mechanism makes it analytically superior to SBB's turn-off response, while TBA's higher association constant and environmental validation give it stronger translational credentials.

**Fig. 13:**
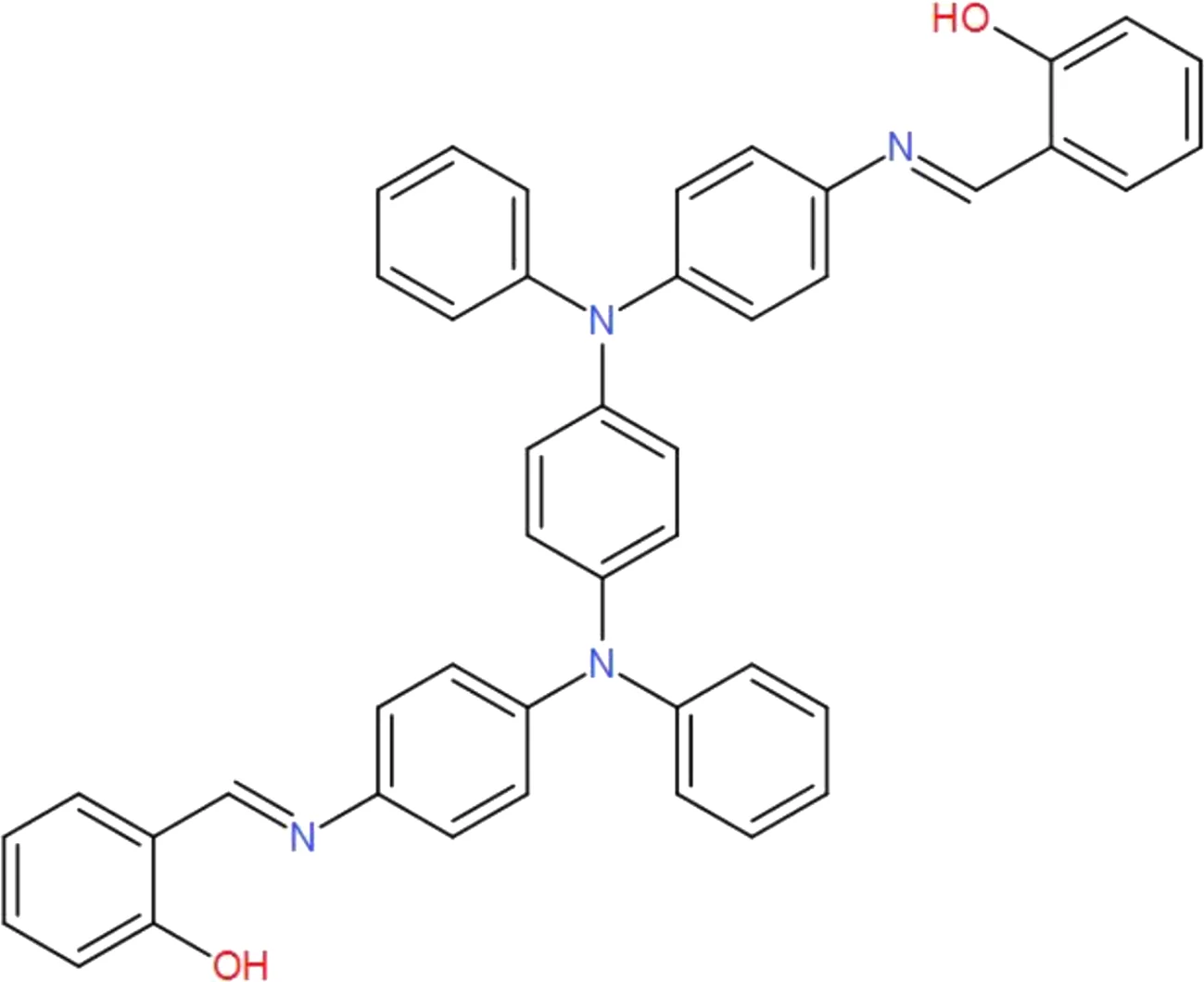
Triphenylamine-based bis-Schiff base sensor TPA for hydrazine detection *via* ICT restriction. Hydrazine coordination at the azomethine bridge restricts intramolecular charge transfer from the triphenylamine donor to the aldehyde acceptor, producing fluorescence quenching and a yellow-to-light-blue colour change under UV; LOD 55.1 nM; applied to HeLa cell imaging.

**Fig. 14:**
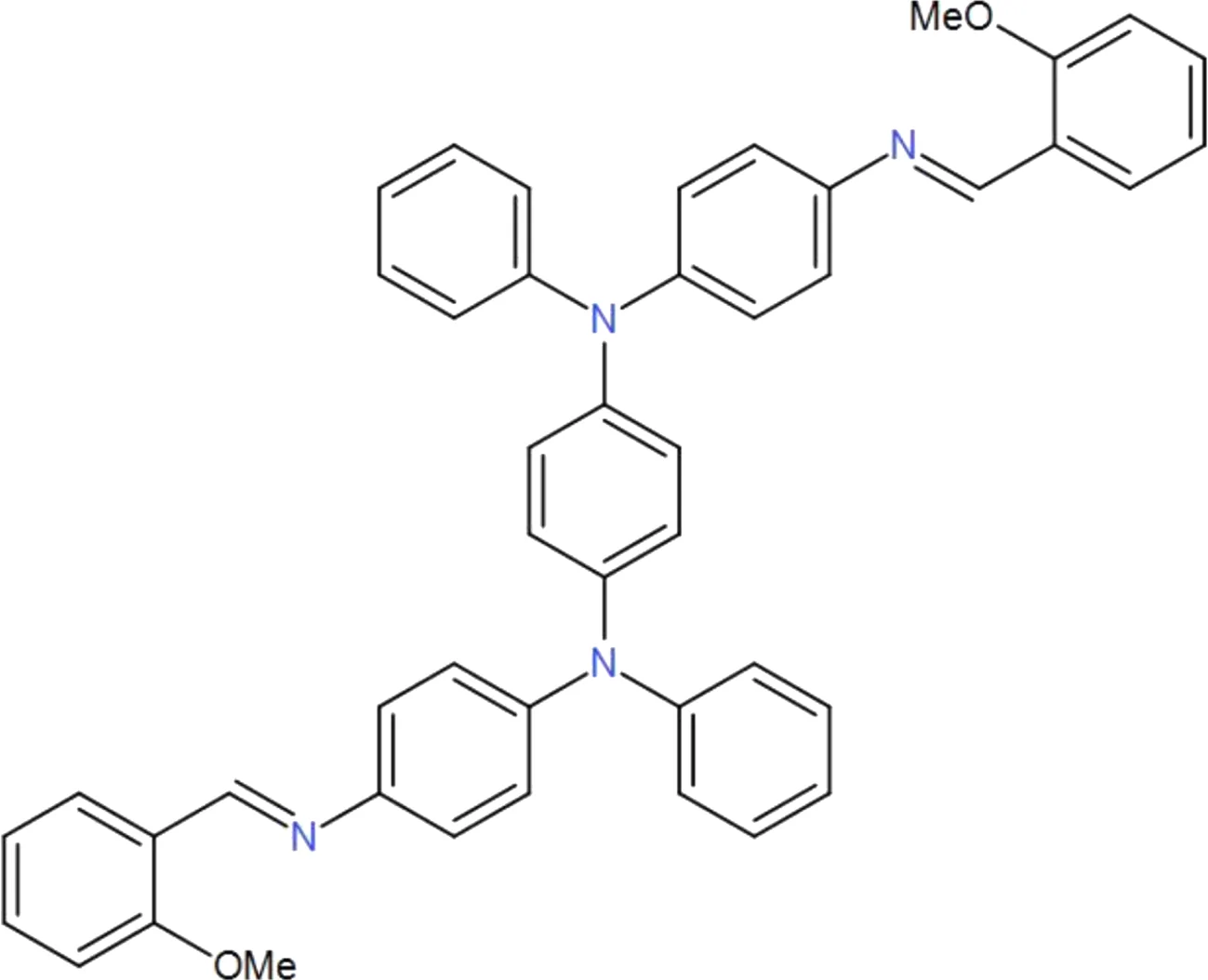
Triphenylamine-based bis-Schiff base sensor TPB for hydrazine detection *via* ICT restriction; identical sensing mechanism to TPA (LOD 55.1 nM), with peripheral structural variation confirming that detection performance is governed by the shared TPA–azomethine scaffold.

A mechanistically distinct strategy exploits ICT modulation through analyte-induced changes in the donor–acceptor electronic architecture. Wang *et al.* synthesised two triphenylamine-based bis-Schiff base fluorescent sensors, TPA and TPB, that detect hydrazine in DMF–water medium through hydrazine-induced restriction of intramolecular charge transfer.^103^ Both probes TPA and TPB, whose structures are shown in Fig. 13 and 14 respectively, feature triphenylamine electron-donor units linked through azomethine bridges to peripheral salicylaldimine terminals, with TPA bearing hydroxyl substituents and TPB bearing methoxy substituents on the terminal aromatic rings. Hydrazine coordinates at the imine bond, increasing molecular rigidity and altering the electronic distribution across the D–A framework, which simultaneously restricts ICT and enhances molecular solubility, resulting in fluorescence quenching with a visible colour change from yellow to light blue under a UV lamp.

Both probes exhibited identical LODs of 55.1 nM, confirming that the sensing performance is governed by the shared triphenylamine–azomethine scaffold rather than the peripheral substituents that distinguish TPA from TPB. A notable strength of this system is the demonstrated applicability in HeLa cell lines, confirming cellular uptake and biocompatibility.

The predominant limitation is the use of an organic solvent co-mixture and the turn-off detection mode, both of which constrain practical biological deployment. Comparison with TBA and SBB reveals that while TPA/TPB achieve a slightly lower LOD than TBA, TBA's aqueous compatibility and genuine environmental sample validation represent a more significant analytical advance than marginally lower detection limits.

**Fig. 15:**
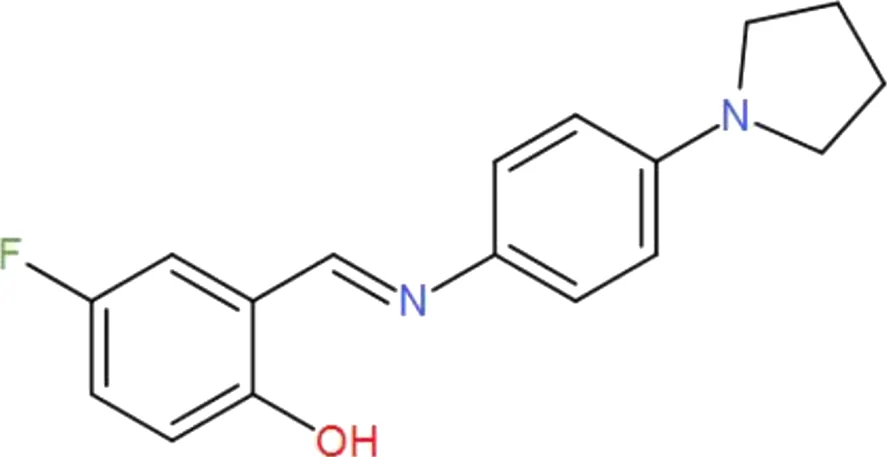
Structure of salicylaldehyde-based Schiff base probe PBAS for dual-mode hydrazine detection integrating ESIPT and AIEE mechanisms. The *ortho*-hydroxyl group positioned adjacent to the imine nitrogen enables ESIPT through enol-to-keto tautomerisation, providing a large Stokes shift, while aggregation of the probe in aqueous media confers AIEE character through restriction of intramolecular rotation. Hydrazine condensation at the imine nitrogen modulates ICT, producing fluorescence turn-on with a yellow-to-colourless colorimetric change and blue-shifted UV absorption at 367 nm; applied to live-cell imaging.

Xie *et al.* developed a salicylaldehyde-based Schiff base probe (PBAS) that integrates both aggregation-induced emission (AIEE) and excited-state intramolecular proton transfer (ESIPT) characteristics for hydrazine detection.^104^ The PBAS probe, whose structure is shown in Fig. 15, incorporates a phenolic –OH group positioned *ortho* to the imine nitrogen, enabling ESIPT through intramolecular proton transfer from the enol to the keto tautomer. The aromatic substituents also act as molecular rotors, imparting AIEE behaviour through restriction of intramolecular rotation upon aggregation. The probe responded selectively to hydrazine through a fluorescence turn-on mechanism accompanied by a distinct colorimetric change from yellow to colourless. UV-visible analysis revealed a blue shift and a decrease in the absorption intensity at 367 nm following hydrazine addition. This behaviour is consistent with hydrazine-induced condensation at the aldehyde terminus, which alters the electron-donating ability of the recognition arm. Consequently, both the ESIPT proton-transfer pathway and the ICT character of the conjugated system are modulated. The combined AIEE–ESIPT architecture offers two complementary analytical advantages. The large intrinsic Stokes shift arising from ESIPT reduces spectral overlap with the excitation light. In addition, the AIEE effect ensures strong fluorescence in aqueous suspension without the aggregation-caused quenching (ACQ) commonly observed in conventional fluorophores. The probe demonstrated high selectivity and was successfully applied to hydrazine imaging in living cells. Among the Schiff base hydrazine probes reviewed, PBAS represents the most photophysically sophisticated design, leveraging three distinct emission-enhancement principles within a single molecular framework.

**Fig. 16:**
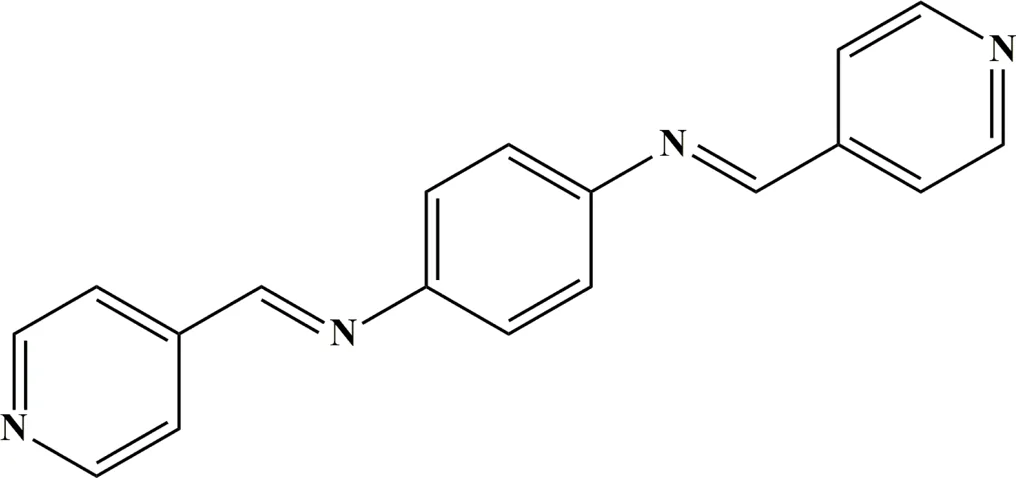
Structure of bis-Schiff base probe S6 for dual-mode hydrazine detection in solution and vapour phase. The two imine nitrogen sites engage hydrazine cooperatively through hydrogen bonding (1 : 2 stoichiometry, *K*_a_ = 1.63 × 10^5^ M^−1^), suppressing PET and producing fluorescence enhancement with yellow-to-colourless colour change; LOD 3.2 ppb; pH range 4–10; vapour-phase detection demonstrated on solid-state films for industrial monitoring.

While solution-phase detection dominates the hydrazine sensor literature, the practical challenge of industrial and workplace monitoring demands solid-state and vapour-phase capable platforms. Ghorai *et al.* reported a bis-Schiff base probe (S6) that achieves dual-mode colorimetric and fluorometric detection of hydrazine in both solution and vapour phases across a broad pH range of 4–10, making it unique among the probes surveyed in this section.^105^ The bis-pyridyl Schiff base probe S6, whose structure is shown in Fig. 16, provides two imine recognition sites that engage hydrazine through cooperative hydrogen bonding in a 1 : 2 binding stoichiometry, confirmed by Job's plot and ^1^H NMR titration, with a high association constant of 1.63 × 10^5^ M^−1^ and a remarkable LOD of 3.2 ppb for solution-phase detection. Hydrazine binding suppresses PET from the lone pairs of the imine nitrogens. This results in enhanced fluorescence together with a yellow-to-colourless colour change that enables naked-eye detection.

The vapour-phase applicability, which is demonstrated by exposure of a solid-state film of S6 to hydrazine vapour, is particularly significant for industrial safety monitoring applications where gaseous emissions must be detected in real time without sample preparation. The main limitation is that hydrogen-bonding-driven recognition is intrinsically less selective than covalent reaction-based approaches in matrices containing other H-bond donors.

**Fig. 17:**
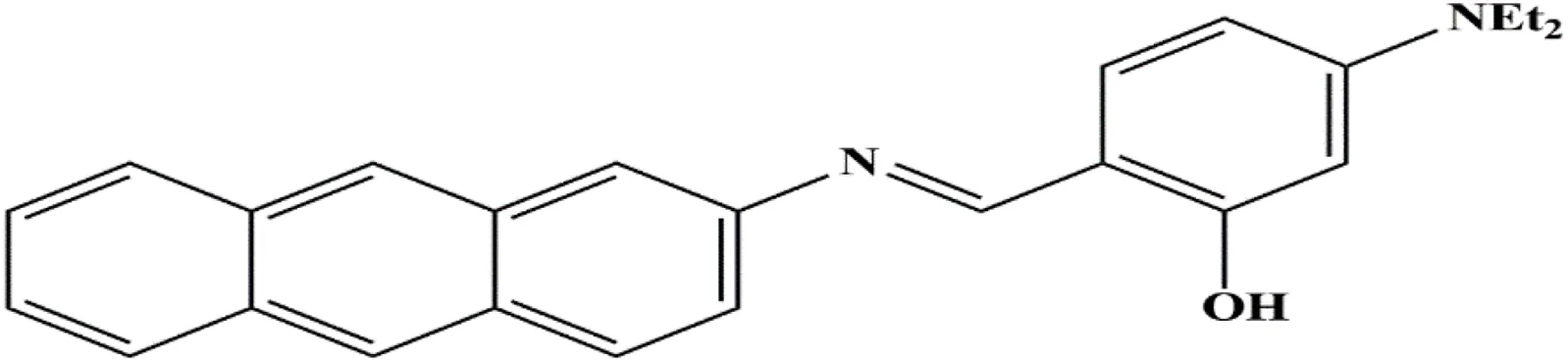
Donor–acceptor Schiff base AIEE active fluorophore S7 for ultrasensitive hydrazine detection *via* aggregation-induced emission. Hydrazine condensation at the imine unit modulates RIR-driven emission; LOD 0.5 ppb in 1 : 1 MeCN–H_2_O; validated in water, urine, mammalian cell imaging, and plant root imaging.

**Fig. 18:**
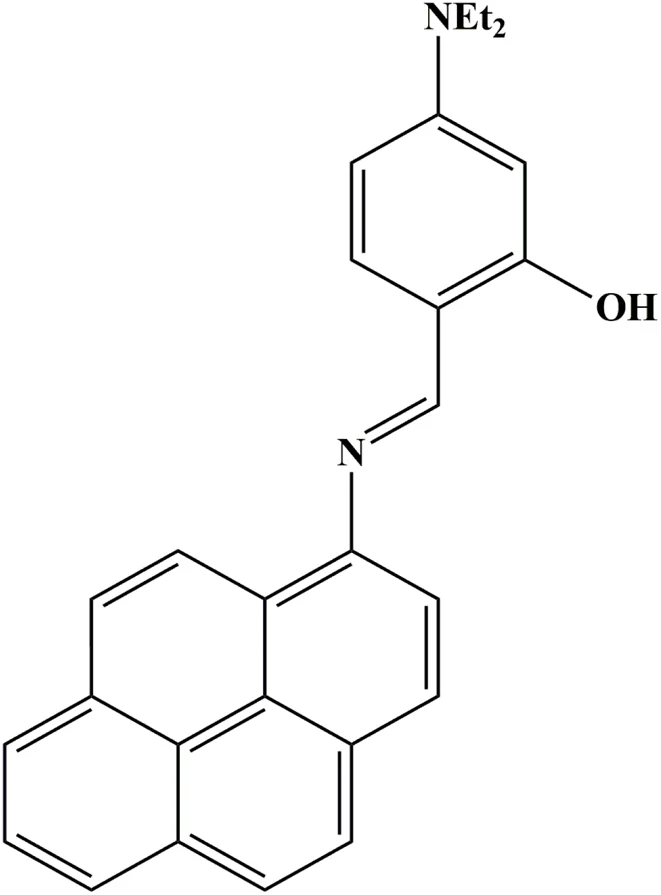
Donor–acceptor Schiff base AIEE active fluorophore S8 for ultrasensitive hydrazine detection; LOD 0.4 ppb (the lowest among surveyed Schiff base hydrazine sensors); dual solution and vapour-phase detection; multi-matrix validated in water, urine, living cells, and plant roots.

A significant advance in sensitivity was achieved by Halder *et al.* who developed two donor–acceptor Schiff base AIEEgens, S7 and S8, whose structures are shown in Fig. 17 and 18 respectively, for ultrasensitive hydrazine detection in both solution and vapour phases.^106^ Both probes feature donor–acceptor electronic asymmetry that promotes strong fluorescence through restriction of intramolecular rotation (RIR) in the aggregated state. Hydrazine reacts with the imine unit by nucleophilic condensation, thereby modulating the emission intensity. The achieved LODs of 0.5 ppb (S7) and 0.4 ppb (S8) in 1 : 1 acetonitrile–water represent the lowest detection limits among the Schiff base hydrazine sensors reviewed, and are well within the regulatory threshold of 10 ppb established by the U.S. EPA. A notable strength of this work is its comprehensive validation across multiple sample types. Both probes were successfully applied to hydrazine detection in water samples and urine, as well as fluorescence imaging in mammalian cells and plant roots. This broad validation demonstrates their potential for environmental, biological, and agricultural applications. This breadth of validation is rare in the Schiff base sensing literature and substantially strengthens the translational case for these probes. The primary caveats are performance dependence on specific aggregation conditions, which may vary with ionic strength and temperature, and the absence of long-term stability data.

Analysis of Table 3 reveals an inverse relationship between mechanistic simplicity and the breadth of real-sample validation. The simplest probe, SBB, has been evaluated only in laboratory water samples, whereas the AIEE-based probes S7 and S8 demonstrate the broadest multi-matrix applicability. This observation suggests that AIEE-based architectures are better suited for complex matrices because their aggregation-state emission is less susceptible to molecular-level interferences than that of single-fluorophore turn-off systems. A second non-trivial trend is the systematic improvement in LOD along the mechanistic progression from H-bonding (SBB: 10^−8^ M), through PET-inhibition (TBA: 10^−7^ M), to ICT-modulation (TPA/TPB: 55 nM), and finally to AIEE-based (S7/S8: 0.4–0.5 ppb). This improvement is not coincidental; it reflects increasing signal amplification per binding event as the mechanisms become more disruptive to the photophysical state of the fluorophore. Third, the comparison between thiophenol (SBMD, LOD 15 nM) and hydrazine probes (best: S7/S8 at 0.4–0.5 ppb) reveals that thiophenol detection achieves lower molar detection limits, attributable to the inherent signal amplification of the p*K*_a_ shift mechanism, whereas hydrazine detection achieves lower mass detection limits owing to its lower molecular weight and the strong driving force for nucleophilic condensation. Notably, the only probe validated in both environmental water and biological matrices is TBA (tap water + river water), suggesting a gap in the literature where hydrazine probes with cellular imaging capability are rarely tested in environmental matrices and *vice versa*. Bridging this gap through dual-matrix validation represents a straightforward but impactful direction for future work.

**Table 3:** Comparative summary of Schiff base-based sensing systems for thiophenol and hydrazine

Probe	Analyte	Detection strategy	Mechanism	LOD	Solvent system	Real sample validation	Key limitation	Reversibility/reusability
SBMD^97^	Thiophenol	Dual-mode (colorimetric + fluorescence)	SNAr cleavage of DNBS; p*K*_a_ shift; merocyanine turn-on	15 nM	Aqueous-compatible	Live-cell imaging	Irreversible SNAr; potential interference from strong nucleophiles	Irreversible (SNAr reaction; chemodosimeter)
SBB^101^	Hydrazine	Dual-mode (colorimetric + fluorescence)	H-bonding at imine; turn-off; isosbestic conversion	9.77 × 10^−8^ M	Mixed organic–aqueous	Water samples	Turn-off mode; limited selectivity *via* H-bonding	Not demonstrated
TBA^102^	Hydrazine	Fluorescence	PET inhibition at imine; turn-on	1.05 × 10^−7^ M	Aqueous solution	Tap water; Minjiang River	Interference studies limited	Not demonstrated
TPA/TPB^103^	Hydrazine	Dual-mode (colorimetric + fluorescence)	ICT restriction *via* azomethine coordination; turn-off	55.1 nM	DMF–H_2_O	HeLa cell imaging	Organic solvent dependence; turn-off mode	Not demonstrated
PBAS^104^	Hydrazine	Dual-mode (colorimetric + fluorescence)	AIEE + ESIPT dual mechanism; turn-on colorimetric	Not specified	Aqueous-compatible (AIEE)	Live-cell imaging	Quantitative LOD not reported; complex photophysics	Not demonstrated
S6 (ref. 105)	Hydrazine (solution + vapour)	Dual-mode (colorimetric + fluorescence)	H-bonding-driven PET suppression; colorimetric + fluorometric	3.2 ppb	Solution + solid film	Industrial vapour monitoring	H-bond selectivity limited in competitive matrices	Reversible (non-covalent H-bonding)
S7/S8 (ref. 106)	Hydrazine (solution + vapour)	Fluorescence	AIEE; nucleophilic condensation at imine; RIR-based turn-on	0.5 ppb (S7); 0.4 ppb (S8)	1 : 1 MeCN–H_2_O	Water, urine, mammalian cells, plant roots	Aggregation-state dependence; long-term stability not studied	Not demonstrated

Across the thiophenol and hydrazine sensing systems reviewed, several recurring analytical limitations define the current state of the field. The most significant limitation is the widespread use of irreversible reaction-based mechanisms, particularly SNAr for thiophenol and imine condensation for hydrazine. Although these reactions provide excellent selectivity and sensitivity, they prevent probe regeneration and dynamic monitoring. This irreversibility also introduces quantitative uncertainty in samples where analyte concentration fluctuates over time, as is common in industrial effluent or intracellular environments. A second limitation is the widespread reliance on mixed organic–aqueous solvent systems. Among the hydrazine probes, only TBA and PBAS operate effectively in pure aqueous media or biologically relevant buffer systems. This solvent dependence directly limits clinical and environmental field deployability. Third, the almost universal absence of long-term stability data, competitive interference studies with other nucleophiles, and reversibility assessments represents a critical gap between analytical demonstration and practical application.

Looking forward, the most productive directions for advancing Schiff base-based thiophenol and hydrazine sensing are as follows. For thiophenol sensing, reversible coordination-based strategies employing soft metal centres such as Au(i) or Pd(ii) represent a promising alternative to irreversible SNAr reactions. These metals form reversible M–S bonds with aromatic thiols while maintaining high selectivity over aliphatic thiols. For hydrazine sensing, the development of ratiometric probes would improve quantitative accuracy in complex matrices. Monitoring wavelength shifts instead of fluorescence intensity would also minimize artefacts caused by photobleaching and variations in probe concentration. For both analyte classes, integrating Schiff base sensing elements into paper strips, electrospun fibre mats, or smartphone-compatible portable devices would facilitate on-site industrial safety monitoring and environmental surveillance. Finally, a systematic computational approach to selectivity engineering, using DFT and TD-DFT calculations to predict the differential reaction kinetics of the sensing element toward the target *versus* competing nucleophiles, would accelerate the rational discovery of probes with improved selectivity profiles.

This section has surveyed eight Schiff base-derived fluorescent and colorimetric chemosensors for two reactive small molecule targets: thiophenol and hydrazine. The SBMD probe demonstrates that merocyanine-based Schiff base systems can achieve nanomolar thiophenol detection with intrinsic ArSH/RSH selectivity through the p*K*_a_ shift mechanism, with demonstrated live-cell imaging capability. For hydrazine, a progression from simple H-bonding (SBB) and PET-inhibition (TBA) systems, through ICT-modulation (TPA/TPB) and dual AIEE–ESIPT (PBAS) architectures, to advanced AIEE-based systems (S6, S7/S8) reveals a clear correlation between mechanistic sophistication and analytical performance, with S7/S8 achieving sub-ppb detection limits validated across four matrix types. The consolidated analysis establishes that AIEE-based Schiff base architectures currently offer the best combination of sensitivity, aqueous compatibility, and multi-matrix applicability among the surveyed probe classes, and that the primary frontier for advancing this field lies in reversibility, aqueous operability, and real-world matrix validation rather than further reductions in detection limits.

### 2.3 Electron transfer-mediated detection of nitroaromatic compounds

Nitroaromatic compounds (NACs) constitute a chemically diverse class of electron-deficient aromatic analytes whose detection has significant implications for both environmental monitoring and counter-terrorism security applications. This class includes not only the nitrophenols and nitrotoluenes that have been most extensively studied using Schiff base probes but also more potent explosive analytes such as 2,4,6-trinitrotoluene (TNTdptz), 2,4-dinitroluene (DNT), 2,4,6-trinitrophenol (TNP/picric acid), and related compounds. Their detection at trace levels in environmental matrices, including soil, water, and air, is a globally important analytical challenge given their persistence, ecotoxicity, and role as explosive precursors or degradation products.^107,108^ Fluorescence quenching through photoinduced electron transfer (PET) is the predominant sensing mechanism in Schiff base-based NAC detection. For highly planar analytes such as TNP, this process is further enhanced by π–π stacking and inner filter effects. Although turn-off fluorescence is characteristic of nitroaromatic sensing, it presents an important analytical limitation. Decreasing fluorescence signals are more susceptible to interference from background fluorescence, photobleaching, and variations in probe concentration, particularly in complex matrices. Turn-on and ratiometric sensing strategies could substantially improve quantitative precision. Although these approaches have been successfully applied to other analyte classes, they remain underdeveloped for nitroaromatic detection.

**Fig. 19:**
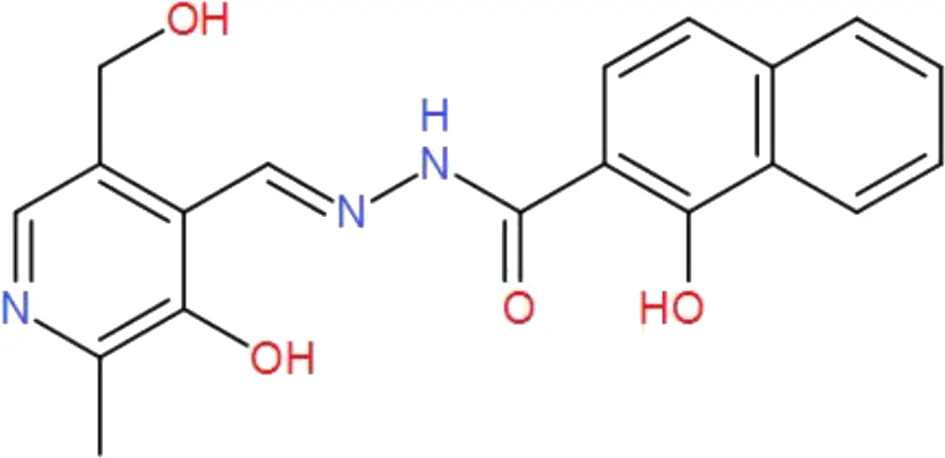
Structure of AIEE-active pyridoxal-derived Schiff base probe NPY for *p*-nitrophenol (*p*-NP) detection. Aggregation of NPY in aqueous media restricts intramolecular rotation of the naphthalene signalling unit, activating AIEE-based emission; addition of *p*-NP induces turn-off fluorescence quenching *via* PET from the electron-rich imine scaffold to the electron-deficient nitrophenol (LOD 1.73 µM); pH-responsive emission adds analytical versatility.

Patel *et al.* developed an AIEE-active pyridoxal-derived Schiff base probe (NPY), whose structure is shown in Fig. 19, for the detection of *p*-nitrophenol (*p*-NP) in water.^107^ The probe exhibits strong aggregation-induced fluorescence enhancement in aqueous media. This AIEE behaviour arises from restriction of intramolecular rotation of the pyridoxal phenyl rings upon aggregation. Addition of *p*-NP produces a turn-off fluorescence response with a LOD of 1.73 µM. The quenching results primarily from PET-driven energy transfer to the electron-deficient nitrophenol, together with a modest inner filter effect. A notable analytical feature of this probe is its pH-responsive emission behaviour, which extends versatility toward environmental sensing applications where pH may vary. However, this pH dependence simultaneously represents an analytical limitation, as the sensing response will vary across different environmental matrices without pH control. The detection limit of 1.73 µM is suitable for industrial wastewater monitoring, where *p*-NP concentrations typically exceed 10 µM. However, it may not satisfy the ultra-trace detection requirements for regulated drinking water, where WHO guideline values are below 1 µM. The practical relevance of NPY would be meaningfully improved by incorporating it into solid-state or paper-based formats that stabilise the aggregation state and reduce pH sensitivity.

**Fig. 20:**
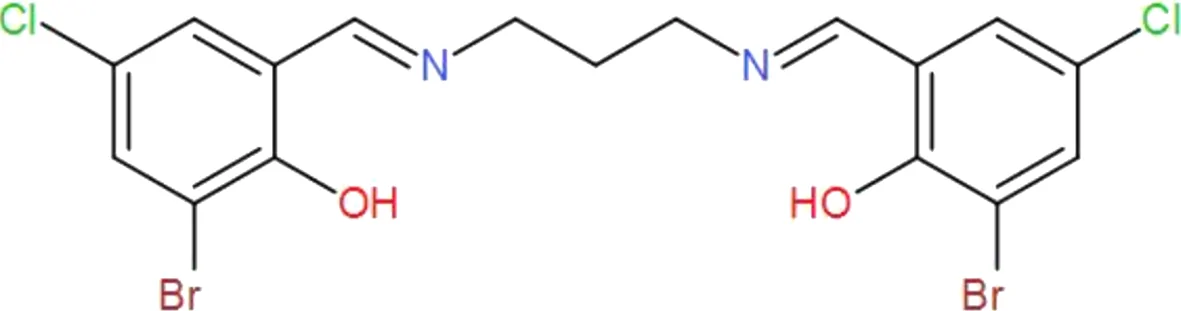
Structure of halogenated bis-Schiff base ligand x-L used to form the zinc complex SBZC (Zn-x-L) for selective 2-nitrophenol (2-NP) detection. The ligand features two imine recognition sites with *ortho*-hydroxyl groups and halogen (Cl, Br) substituents. Zn^2+^ coordination rigidifies the scaffold *via* CHEF, providing high baseline emission; 2-NP-induced turn-off quenching (LOD 2.2 µM) is selective over other nitroaromatic isomers with stable response across solvents of varying polarity.

A structurally distinct approach was demonstrated by Xu *et al.*, who reported a halogenated Schiff base ligand (x-L), whose structure is shown in Fig. 20, that forms a zinc complex (SBZC, Zn-x-L) for the selective detection of 2-nitrophenol (2-NP).^109^ Zn^2+^ coordination serves two important functions. It rigidifies the Schiff base scaffold through chelation, thereby enhancing fluorescence *via* the CHEF mechanism. In addition, it creates a structurally defined coordination cavity that improves selectivity for 2-NP through steric and geometric complementarity. The strong fluorescence quenching response with a LOD of 2.2 µM and impressive stability across solvents of varying polarity confirm that the metal coordination framework provides analytical robustness against environmental variability. Compared with the free Schiff base probes for nitrophenols, the zinc complex strategy offers inherently superior baseline fluorescence (CHEF) and thus a better signal-to-noise ratio for the quenching response. However, as with the other probes this subsection, the turn-off detection mode limits quantitative precision in turbid or coloured matrices, and the LOD does not meet the ultra-trace requirements for contaminated groundwater analysis. Integration into a ratiometric probe design through introduction of a second emission-reporting unit into the Zn complex would represent a straightforward advance.

**Fig. 21:**
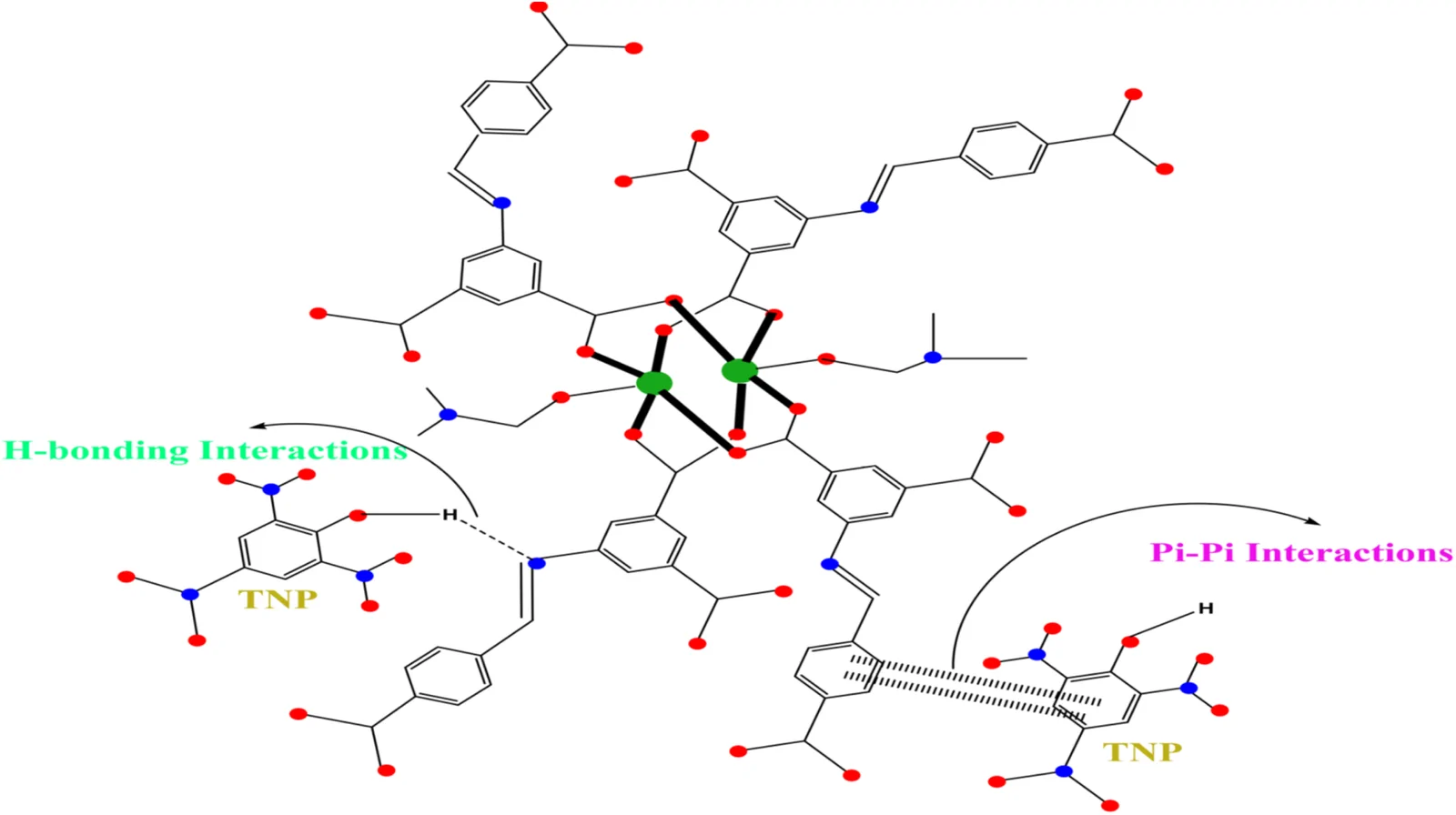
Copper-based Schiff base tricarboxylate MOF for aqueous TNP (picric acid) detection. The rigid MOF framework prevents imine hydrolysis while providing an extended conjugated sensing scaffold; TNP-induced PET and π–π stacking quenching gives a Stern–Volmer constant of 1.07 × 10^4^ M^−1^ and LOD 80 ppb; DFT studies confirmed the electron transfer mechanism; good water stability demonstrated.^110^ Adapted/reproduced from ref. 110 with permission from Springer Nature,^110^ Copyright 2021.

Kaur *et al.* extended Schiff base-based NAC sensing into the metal–organic framework (MOF) domain by constructing a copper-based MOF from a Schiff base tricarboxylate ligand for the selective detection of 2,4,6-trinitrophenol (TNP, picric acid) in aqueous media, as depicted in Fig. 21.^110^ The MOF platform enhances the sensing response through two complementary mechanisms. The rigid framework provides high intrinsic fluorescence and a large surface area for analyte adsorption. In addition, the strong electron-withdrawing nature of TNP promotes efficient PET and static quenching through π–π interactions within the porous network. The resulting Stern–Volmer constant of 1.07 × 10^4^ M^−1^ and LOD of 80 ppb for TNP represent among the strongest sensing performances in this analyte class, and the demonstrated water stability and structural robustness of the MOF distinguish it from molecular probes that suffer from imine hydrolysis under aqueous conditions. Among the probes reviewed in Section 2.3, the MOF platform is the only one that fundamentally addresses the aqueous stability limitation through structural immobilisation of the imine within a crystalline framework, a design principal worthy of broader adoption. However, the selectivity for TNP among multiple nitroaromatics, while high, requires rigorous validation in field matrices containing complex nitroaromatic mixtures typical of explosive-contaminated soil or water.

**Scheme 2:**
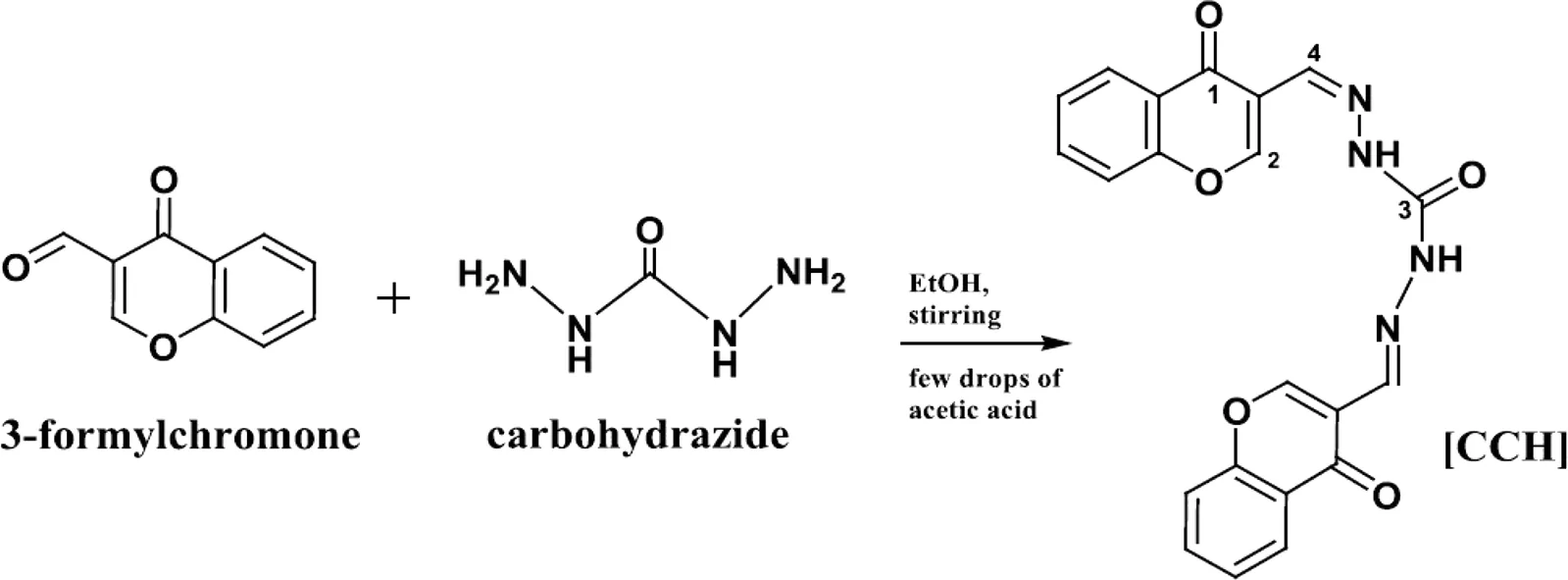
Synthesis of chromone-derived Schiff base probe CCH from 3-formylchromone and carbohydrazide in ethanol with catalytic acetic acid. The probe achieves selective 1 : 1 binding with *p*-nitrotoluene (*K*_a_ = 1.2 × 10^5^ M^−1^) with fluorescence quenching at 402 nm (LOD 25 nM); mechanism confirmed by DFT, NMR, and HRMS.^111^ Adapted/reproduced from ref. 111 with permission from Elsevier,^111^ Copyright 2022.

Tomer *et al.* reported a chromone-derived Schiff base probe (CCH), synthesised from 3-formylchromone and carbohydrazide as shown in Scheme 2, for the selective detection of *p*-nitrotoluene (*p*-NT).^111^*p*-NT is a precursor and environmental degradation product of TNT and its selective detection over structurally similar nitroaromatics is analytically challenging. CCH achieves this selectivity through its tight 1 : 1 binding interaction with *p*-NT, confirmed by Job's plot and HRMS analysis, with an exceptionally high association constant of 1.2 × 10^5^ M^−1^ and a remarkably low LOD of 25 nM. The pronounced fluorescence quenching at 402 nm with minimal interference from other nitroaromatics establishes strong structural selectivity. Notably, among all the nitroaromatic probes reviewed, CCH achieves the lowest LOD (25 nM), approximately one order of magnitude below the TNP MOF (80 ppb), demonstrating that molecular probes can outperform MOF platforms for specific analyte–probe pairs where binding geometry is precisely complementary. The dual functionality of CCH for both Cu^2+^ ion detection and *p*-NT sensing within a single scaffold is also architecturally notable, illustrating the potential for Schiff base probes to address multiple detection needs simultaneously. Future translation would benefit from real sample validation in explosive-contaminated matrices.

**Fig. 22:**
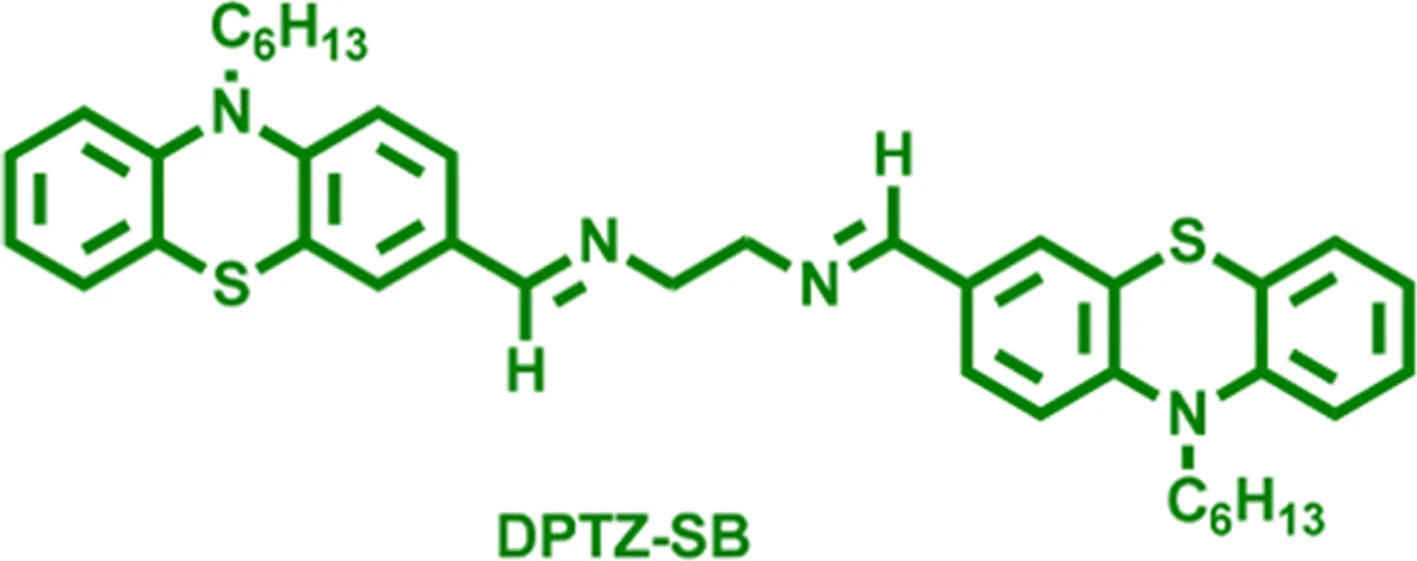
Structure of phenothiazine-based Schiff base AIEEgen DPTZ-SB for picric acid (PA) detection. The two phenothiazine wings flanking the central ethylenediamine-bis-imine scaffold provide AIEE activity through restriction of intramolecular rotation (RIR) in aqueous media (9.4-fold emission enhancement); PA quenching proceeds via PET and inner filter effect (LOD 0.2 ppb, *K*_a_ = 4.05 × 10^1^ M^−1^); validated in real water samples via portable paper-based strip with smartphone-assisted colorimetric readout.^112^ Adapted/reproduced from ref. 112 with permission from Elsevier,^112^ Copyright 2023.

Barot *et al.* developed a phenothiazine-based Schiff base probe (DPTZ-SB), whose structure is shown in Fig. 22, for the detection of picric acid (PA).^112^ DPTZ-SB exhibits strong AIEE behaviour with a 9.4-fold fluorescence enhancement in aqueous media, arising from restricted intramolecular rotation of the phenothiazine wings upon aggregation. PA detection proceeds through a combination of PET from the electron-rich phenothiazine donor to PA and inner filter effect quenching due to spectral overlap between PA absorption and probe emission. The probe achieved an LOD of 0.2 ppb and a binding constant of 4.05 × 10^1^ M^−1^, representing the best analytical performance among the probes summarised in Table 4. Furthermore, successful validation in real water samples using a portable paper-based platform with smartphone-assisted quantitative readout (Fig. 22) demonstrates its strong practical potential. The paper-strip format addresses the real-world deployment requirement for explosive detection directly: a field analyst or security officer can use the device without laboratory equipment, which is the ultimate goal for security monitoring. The primary remaining gap is long-term stability data for the paper-based device under storage and field conditions, which is essential before deployment in security applications.

**Table 4:** Comparative summary of Schiff base-based nitroaromatic compound sensors

Probe	Analyte	Detection strategy	Mechanism	LOD	Solvent	Real sample	Key limitation	Reversibility/reusability
NPY^107^	*p*-NP	Fluorescence	AIEE + PET quenching	1.73 µM	Aqueous	Wastewater (proposed)	pH dependence; turn-off; no field validation	Not demonstrated
SBZC^109^	2-NP	Fluorescence	CHEF baseline + PET turn-off	2.2 µM	Multi-solvent stable	Not demonstrated	Turn-off mode; LOD insufficient for drinking water	Not demonstrated
MOF (Cu-Schiff base)^110^	TNP	Fluorescence	PET + π–π stacking quenching in MOF pores	80 ppb	Aqueous	Environmental water (aqueous stability shown)	Turn-off; selectivity in mixed NAC matrices not validated	Partially reversible (MOF structurally regenerable)
CCH^111^	*p*-NT	Fluorescence	Static quenching; 1 : 1 binding	25 nM	Mixed solvent	Not demonstrated	Turn-off; no real explosive matrix validation	Not demonstrated
DPTZ-SB^112^	PA (picric acid)	Dual-mode (colorimetric + fluorescence)	AIEE + PET + inner filter effect	0.2 ppb	Aqueous	Real water; paper strip + smartphone	Turn-off; paper device long-term stability not studied	Not demonstrated

Beyond the representative probes summarized in Table 4, recent studies published in 2026 further illustrate the continued evolution of Schiff base-based neutral-analyte sensing. Notably, an ESIPT/AIEE-active Schiff base fluorophore enabled highly sensitive detection of picric acid and ammonia at nanomolar concentrations.^113^ The probe also demonstrated practical utility in anticounterfeiting and latent fingerprint visualization. Mechanistic studies of Schiff base ESIPT–AIEE systems have provided deeper insight into the factors governing their photophysical behaviour.^114^ These studies revealed that molecular packing, intermolecular interactions, and restriction of intramolecular motion play crucial roles in controlling fluorescence emission and analyte responsiveness in the aggregated state. Such findings complement earlier reports on AIEE- and ESIPT-based Schiff base probes for neutral targets, including hydrazine, biothiols, nitroaromatic explosives, and volatile analytes. These systems benefit from large Stokes shifts, reduced background interference, and improved performance in aqueous and solid-state environments. Collectively, recent advances indicate a clear trend towards multifunctional ESIPT–AIEE platforms with enhanced sensitivity, solid-state operability, and practical applicability. These developments continue to broaden the scope of Schiff base chemosensors for environmental monitoring, bioimaging, security screening, and related neutral-analyte sensing applications.

Analysis of the five probes in Table 4 reveals that overall analytical performance broadly correlates with structural sophistication: molecular probes with AIEE character (NPY, DPTZ-SB) and MOF-based systems achieve lower LODs and better aqueous compatibility than simple ICT-based fluorophores. However, the universal reliance on fluorescence quenching (turn-off mode) across all five systems is a fundamental analytical limitation that the field has not yet adequately addressed. In a quenching-based sensor, any source of probe concentration variability, background matrix fluorescence, or photobleaching produces an apparent quenching signal that is analytically indistinguishable from a genuine analyte response. For security-critical applications such as explosive residue detection in soil or forensic swabs, false-positive responses carry severe practical consequences, making turn-on or ratiometric detection strongly preferable. The CCH probe comes closest to addressing selectivity concerns through its exceptionally high association constant, but still operates in turn-off mode. The TNP MOF is the only system that inherently addresses imine hydrolysis through structural immobilisation, but its selectivity profile in complex nitroaromatic mixtures has not been rigorously validated. Real-sample testing in explosive-contaminated environmental matrices, including field soil, water, and surface swabs, is largely absent from all reported systems and represents the most critical gap between laboratory demonstrations and practical deployment.

Future Schiff base-based NAC sensors should prioritize turn-on or ratiometric detection mechanisms. Broader validation in real-world environmental matrices will also be essential for competing with established HPLC–MS and immunoassay methods.

### 2.4 Detection of neutral organic molecules

The optical detection of neutral organic molecules presents fundamentally different design challenges compared to ionic or reactive analyte sensing. Without the benefit of direct electrostatic or coordination driving forces, probe–analyte binding must rely on weaker non-covalent forces, hydrogen bonding, van der Waals interactions, host–guest complementarity, or covalent reaction-based mechanisms specifically engineered to exploit structural features of the target molecule. This section covers Schiff base-based sensing of environmentally significant herbicides (mesotrione and Starane), biologically important carbonyl compounds (formaldehyde and related aldehydes), chlorinated VOCs, and the analytically unusual case of salicylaldehyde as a reaction-based probe target.

**Fig. 23:**
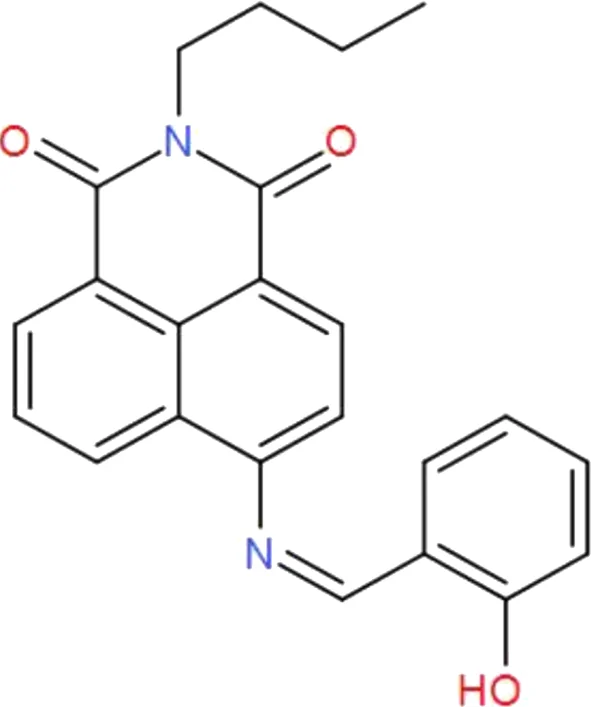
Schiff base fluorescent probe HNA for mesotrione herbicide detection. Mesotrione's 1,3-diketone moiety undergoes nucleophilic attack on the imine carbon of HNA, cleaving the C=N bond and releasing a fluorescent naphthalimide amine fragment (turn-on, LOD 0.16 µM); reaction-based mechanism provides chemical selectivity; validated in agricultural and environmental water samples.

Mesotrione is a widely used triketone herbicide for broadleaf weed control in maize cultivation, and its environmental persistence in soil and surface water necessitates sensitive analytical monitoring methods.^115–117^ Liu *et al.* developed a Schiff base fluorescent probe, 6-(2-hydroxybenzylidene)-*N-n*-butyl-naphthalimide (HNA), for the selective detection of mesotrione, as shown in Fig. 23.^118^ The sensing mechanism is based on mesotrione-induced cleavage of the Schiff base C=N bond. The 1,3-diketone moiety of mesotrione attacks the imine carbon, releasing a fluorescent naphthalimide amine fragment. This reaction-based mechanism provides inherent chemical selectivity over non-nucleophilic herbicides and produces a clear turn-on fluorescence response with a LOD of 0.16 µM. Validation in agricultural and environmental water samples confirms practical applicability for mesotrione residue monitoring. The irreversible nature of the C=N cleavage reaction, however, prevents probe recycling, and further evaluation against structurally related triketone herbicides would strengthen the selectivity claim.

Starane (fluroxypyr-meptyl) is a selective hormone herbicide widely used in cereal crops including wheat and rice, with associated concerns for aquatic ecosystem toxicity at trace residue levels.^119^ Wu *et al.*^120^ developed a diamino-naphthalene Schiff base-bridged bis-tetraphenylimidazole (Bis-TPI) fluorescent sensor for starane detection. The Bis-TPI probe exhibits weak green fluorescence in DMSO–water (95% water fraction) because of restriction of intramolecular rotation in the partially aggregated state. Upon binding starane within the macrocyclic cavity, the probe produces a strong blue fluorescence turn-on response. The defined Bis-TPI cavity forms a 1 : 1 host–guest complex with starane, providing both high sensitivity (LOD 0.015 µM) and excellent selectivity. The cavity geometry is complementary to starane but not to structurally different herbicides. Validated application in real water and fresh leaf samples demonstrates broad-matrix applicability. Among the two herbicide probes reviewed, Bis-TPI provides superior analytical performance in terms of detection limit, aqueous compatibility, and multi-matrix validation. These findings demonstrate that host–guest cavity design can achieve sensitivity comparable to that of reaction-based sensing mechanisms. The coverage of Schiff base-based herbicide detection remains limited, with atrazine, glyphosate, and chlorsulfuron representing major herbicide classes for which Schiff base probes have not yet been reported and represent an important gap in the literature.

Formaldehyde (FA) is a Group 1 carcinogen (IARC classification) ubiquitous in indoor environments as a product of combustion, building materials, and food preservation processes, with chronic exposure linked to nasopharyngeal cancer, heart disease, and neurological impairment.^121,122^ Its selective fluorometric detection is analytically challenging because formaldehyde is the simplest carbonyl species and must be distinguished from structurally similar aldehydes including acetaldehyde, benzaldehyde, and glyoxal that coexist in environmental and food matrices. Schiff base chemistry offers a direct approach to formaldehyde sensing. Primary amine-functionalised fluorophores react with FA through condensation to form imine products. This Schiff base formation suppresses PET from the amine to the fluorophore, producing a turn-on fluorescence response. This sensing mechanism distinguishes FA from other analytes through differences in the rate and reversibility of the condensation reaction, which depend on the electrophilicity of the carbonyl group. Four distinct Schiff base-derived probes have been developed for formaldehyde detection, revealing an interesting mechanistic and performance progression.

The DTH probe, based on 2,5-dihydroxy-*p*-benzenedicarbonamide as the fluorophore and hydrazine as the reactive recognition unit, was developed specifically for the simultaneous discrimination of formaldehyde and acetaldehyde.^123^ The critical mechanistic insight of this probe is that the electron-donating properties of the –H substituent (formaldehyde) and the –CH_3_ substituent (acetaldehyde) in the respective imine products FA-DTH and AA-DTH produce measurably different fluorescence quantum yields (0.055 and 0.122, respectively) and emission characteristics. DTH functions as a ratiometric probe for formaldehyde (*R* = 534/508 nm) and as a turn-on probe for acetaldehyde. The emission wavelength shows a linear correlation with the FA/AA molar ratio in mixed solutions. The LOD of 0.29 µM for FA is moderate compared to the other probes reviewed here, but the simultaneous discrimination capability is unique and analytically valuable for complex atmospheric or food matrices where both aldehydes coexist. The probe has been applied to HeLa cells, confirming biocompatibility and cellular uptake.

A substantially enhanced sensitivity was achieved with the naphthalimide-derived probe HBD, which detects formaldehyde through imine formation at a hydrazone recognition unit positioned adjacent to the naphthalimide chromophore.^124^ The naphthalimide fluorophore provides a high-brightness, photostable platform whose emission at 550 nm is sensitive to the electronic state of the recognition arm. HBD achieves LODs of 10.6 nM for FA and 7.3 nM for acetaldehyde. This represents an approximately 27-fold improvement in FA sensitivity over DTH, owing to the high fluorescence quantum yield and molar absorptivity of the naphthalimide platform. The rapid response time of approximately 1 minute, confirmed by kinetic fluorescence monitoring, makes HBD suitable for real-time monitoring applications. However, unlike DTH which discriminates FA from AA by ratiometric response, HBD shows comparable sensitivity to both aldehydes, meaning discrimination requires additional selectivity strategies or separation steps. This probe is particularly well-suited for applications where total aldehyde burden rather than individual species must be quantified.

A third approach, reported by Ye *et al.* using probe NapNH_2_, extended Schiff base aldehyde sensing toward dual-analyte detection of both formaldehyde and phosgene.^125^ NapNH_2_ bears a naphthalene-amine fluorophore that reacts with FA through condensation (LOD 62 nM) and with phosgene through carbamylation of the amine group (LOD 23 nM), with the two reactions producing spectrally distinct fluorescence responses. This dual-analyte design uses the reactive amine as a bifunctional recognition site for two chemically distinct electrophiles. The approach is mechanistically innovative and reflects the growing trend toward multiplexed sensing using a single probe. The selectivity between FA and phosgene arises from the difference in reaction kinetics: phosgene reacts rapidly through nucleophilic acyl substitution while FA reacts through slower condensation, enabling temporal discrimination. The fast response behaviour of NapNH_2_ to both analytes was confirmed by time-resolved fluorescence monitoring.

Finally, a quinolimide-based Schiff base probe reported by Wechakorn *et al.* demonstrated a linear fluorescence response to FA over the concentration range of 0–180 µM with a LOD of 1.7 µM through an NBD (4-nitrobenzo-2-oxa-1,3-diazole)-conjugated ratiometric mechanism with a large Stokes shift.^124^ Although the LOD is the highest among the four formaldehyde probes reviewed (1.7 µM *vs.* 0.29 µM for DTH, 10.6 nM for HBD, 62 nM for NapNH_2_), the exceptionally wide linear range of 0–180 µM is analytically advantageous for monitoring FA in heavily contaminated environments such as embalming facilities or formaldehyde-intensive industrial processes where concentrations far exceed the nanomolar range.

Comparison of the four formaldehyde probes highlights their complementary strengths. HBD provides the highest sensitivity, DTH uniquely discriminates between FA and acetaldehyde, NapNH_2_ enables dual detection of FA and phosgene, and the quinolimide probe offers the widest linear dynamic range. This mechanistic and performance complementarity suggests that the most analytically complete approach would be a sensor array combining multiple Schiff base probes.

**Scheme 3:**
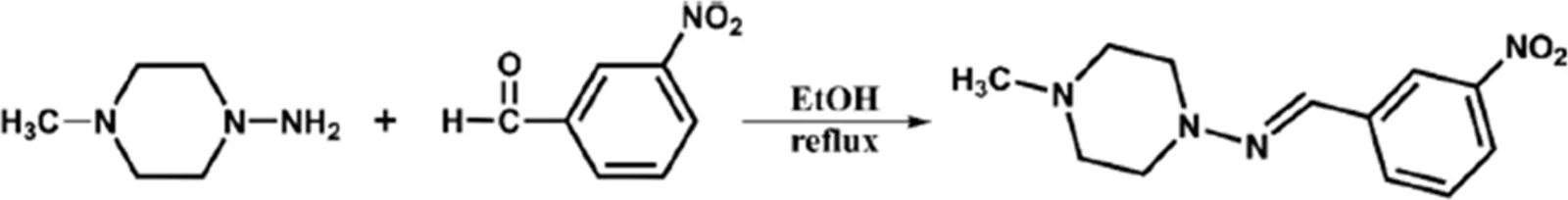
Synthesis of piperazine-based Schiff base PBSB from 4-methylpiperazin-1-amine and 3-nitrobenzaldehyde in ethanol under reflux. The nitrobenzyl electron-withdrawing group promotes intermolecular interactions with electron-deficient chlorinated VOC vapours; dichloromethane LOD 1.85 ppm, LOQ 5.62 ppm (SPR units); reversible response/recovery times 2 s/5 s for real-time vapour monitoring.^130^ Adapted/reproduced from ref. 130 with permission from Springer Nature,^130^ Copyright 2024.

Volatile organic compounds (VOCs) encompass a broad class of carbon-based chemicals with high vapour pressures under ambient conditions, including chlorinated solvents, aromatic hydrocarbons, and aldehyde vapours. Their chronic environmental and occupational exposure is linked to mutagenicity, carcinogenicity, central nervous system toxicity, and respiratory damage, making on-site real-time vapour-phase detection a public health priority.^126–129^ The detection of chlorinated VOCs (CVOCs) presents a particular challenge for molecular fluorescent sensors, as their chemical inertness and low polarity provide few reactive handles for covalent recognition. Halay *et al.*^130^ addressed this challenge by reporting a piperazine-based Schiff base, 4-methyl-*N*-(3-nitrobenzylidene)piperazin-1-amine (PBSB), synthesised as shown in Scheme 3, as a surface plasmon resonance (SPR) optochemical sensor material for the detection of trichloroethylene, dichloromethane, and chloroform vapours. The sensing mechanism relies on polarity-induced optical changes at the Schiff base thin-film surface following vapour adsorption rather than covalent reaction. The electron-withdrawing nitrobenzyl group enhances intermolecular interactions with chlorinated vapours through dipole–dipole and van der Waals forces. The probe exhibited a dichloromethane LOD of 1.85 ppm and LOQ of 5.62 ppm by SPR, with rapid response and recovery times of 2 and 5 seconds respectively, confirming suitability for real-time vapour monitoring in industrial settings. The fast and reversible response is particularly important for workplace safety monitoring, where continuous detection rather than single-measurement analysis is required.

The primary analytical challenges are selectivity in complex vapour mixtures and sensitivity to environmental humidity and temperature, which modulate thin-film adsorption kinetics and must be addressed before field deployment. The solid-state SPR format of PBSB, however, represents a conceptually important advance over solution-phase Schiff base probes by integrating the molecular sensor into a portable optochemical device architecture.

**Scheme 4:**
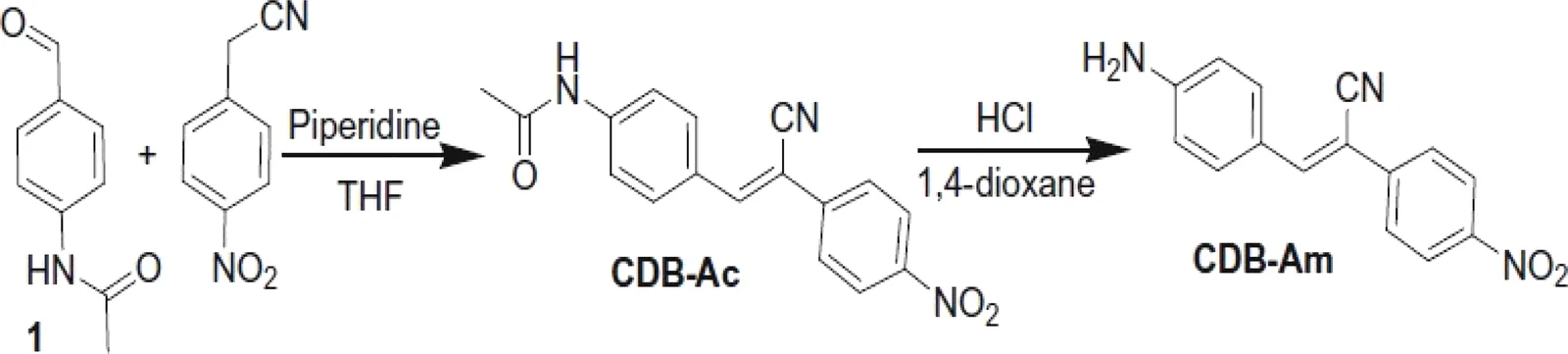
Two-step synthesis of probe CDB-Am from compound 1 via intermediate CDB-Ac. The primary amine of CDB-Am reacts selectively with the aldehyde group of salicylaldehyde to form imine adduct CDB-SA; the *ortho*-hydroxyl group of salicylaldehyde forms an intramolecular H-bond with the imine nitrogen, stabilising the E-configuration and conferring selectivity over other aldehydes; imine formation suppresses PET and restores AIEE emission (LOD 0.94 µM, range 3.1–40 µM).^131^ Adapted/reproduced from ref. 131 with permission from Springer Nature,^131^ Copyright 2021.

Salicylaldehyde (2-hydroxybenzaldehyde) is included in this section as a neutral organic analyte target rather than a metal ion or charged species, though its inclusion in a neutral molecule sensing review warrants explicit justification. Salicylaldehyde is a naturally occurring aromatic aldehyde found in willow bark and various plant species, and its selective detection is relevant to food authenticity assessment, pharmaceutical quality control, and environmental monitoring of phenolic aldehydes in industrial effluents. Analytically, it presents a unique challenge among the analytes covered in this review: being itself an aldehyde, it can function as a Schiff base precursor and form imines with primary amines in the probe. This provides an intrinsic and highly selective covalent recognition mechanism distinct from the coordination or electrostatic binding strategies used for other analytes. Chen *et al.* exploited precisely this chemistry to develop a turn-on fluorescent probe, CDB-Am, whose synthesis is shown in Scheme 4, for salicylaldehyde detection through PET modulation coupled with AIEE enhancement. CDB-Am contains a primary amine recognition unit that reacts selectively with the aldehyde group of salicylaldehyde to form the Schiff base adduct CDB-SA. Formation of the imine suppresses PET from the amine lone pair to the fluorophore, thereby restoring AIEE-based emission.^131^ The *ortho*-hydroxyl group of salicylaldehyde, which is absent in benzaldehyde and aliphatic aldehydes, forms an intramolecular hydrogen bond with the imine nitrogen in CDB-SA. This interaction stabilises the E-imine configuration and provides the structural basis for selectivity. The reaction-based mechanism thus exploits the structural uniqueness of salicylaldehyde rather than relying on weaker affinity-based discrimination, justifying its inclusion as a mechanistically instructive example of aldehyde-specific Schiff base chemistry. The probe achieves a LOD of 0.94 µM over a detection window of 3.1–40 µM, with full structural validation by ^1^H NMR, mass spectrometry, and FTIR. The primary limitations are the relatively narrow concentration window and potential for interference from other *ortho*-hydroxyaldehyde species in complex matrices.

Across the neutral organic molecule sensing systems reviewed, several cross-cutting patterns are evident. Reaction-based probes that exploit covalent imine formation (formaldehyde probes, HNA for mesotrione, CDB-Am for salicylaldehyde) generally achieve lower LODs than non-covalent host–guest systems for the same analyte class, reflecting the signal amplification inherent to chemical transformation rather than binding equilibrium. However, covalent sensing precludes reversibility and sensor reuse. The VOC sensor (PBSB/SPR) represents the only reversible system in this section, as non-covalent van der Waals interactions with the PBSB thin film are inherently reversible, and this reversibility comes at the cost of moderate selectivity. Herbicide coverage remains thin with only two probes reported, and the major commercially significant herbicide classes, including atrazine, chlorpyrifos, and glyphosate, have not been addressed by Schiff base-derived probes. This represents a large and practically important gap given the global scale of herbicide use and the associated groundwater contamination issues.

Comparison of the four formaldehyde probes suggests that a sensor array combining complementary linear ranges, discrimination capabilities, and sensing mechanisms would provide superior analytical performance compared with any individual probe.

Table 5 reveals several mechanistically significant trends in Schiff base-based sensing of neutral organic molecules. The most notable is the strong correlation between reaction-based sensing mechanisms and low detection limits. Probes that rely on covalent imine formation or C–N bond cleavage, including HNA (mesotrione, LOD 0.16 µM), DTH (formaldehyde, LOD 0.29 µM), and Bis-TPI (Starane, LOD 0.015 µM), consistently achieve nanomolar-to-low-micromolar sensitivity that is suitable for environmental monitoring. This outperforms the non-covalent host–guest and polarity-sensitive systems in the same table, confirming that the signal amplification inherent in chemical transformation at the imine recognition site is the most powerful design strategy for neutral analyte detection with Schiff base probes.

**Table 5:** Comparative summary of Schiff base-based neutral organic molecule sensors

Probe	Analyte	Detection strategy	Mechanism	LOD	Solvent system	Key strength	Key limitation	Reversibility/reusability
HNA^118^	Mesotrione	Fluorescence	C=N bond cleavage by 1,3-diketone; turn-on	0.16 µM	Mixed solvent	Reaction-based; validated in agri/env water	Irreversible; no recyclability	Irreversible (C=N cleavage; chemodosimeter)
Bis-TPI^120^	Starane	Fluorescence	Host–guest cavity; turn-on	0.015 µM	DMSO–H_2_O (95%)	Excellent sensitivity; real water and leaf validated	Specific cavity; limited analyte scope	Reversible (non-covalent host–guest cavity)
DTH^82^	FA/AA	Fluorescence	Imine formation; ratiometric FA; turn-on AA	0.29 µM (FA)	PBS buffer	Unique FA/AA discrimination; cell imaging	Moderate sensitivity; irreversible	Irreversible (imine formation; chemodosimeter)
HBD^124^	FA/AA	Fluorescence	Hydrazone Schiff base; PET inhibition; turn-on	10.6 nM (FA)	Aqueous	Best sensitivity among FA probes; fast (1 min)	No FA/AA discrimination; irreversible	Irreversible (hydrazone formation; chemodosimeter)
NapNH_2_ (ref. 124)	FA + phosgene	Dual-mode (colorimetric + fluorescence)	Condensation (FA) and carbamylation (phosgene); dual	62 nM (FA); 23 nM (phosgene)	Aqueous	Dual-analyte detection; temporal discrimination	Selectivity in complex mixed matrices not fully validated	Irreversible (condensation + carbamylation)
Quinolimide-NBD^125^	FA	Fluorescence	Ratiometric imine formation; large Stokes shift	1.7 µM	Mixed solvent	Wide linear range (0–180 µM); ratiometric	Lowest sensitivity among FA probes	Irreversible (imine formation; chemodosimeter)
PBSB^130^	CVOCs (DCM, TCE, CHCl_3_)	Colorimetric (SPR-based)	Polarity-induced SPR thin-film adsorption; reversible	1.85 ppm (DCM)	Solid-state vapour phase	Reversible; fast (2 s); portable SPR format	Limited selectivity in mixed VOC environments	Reversible (van der Waals adsorption; 5 s recovery)
CDB-Am^131^	Salicylaldehyde	Fluorescence	*In situ* Schiff base formation; PET suppression + AIEE	0.94 µM	Aqueous	Mechanistically selective; AIEE; structurally validated	Narrow detection window; *ortho*-hydroxy interference possible	Irreversible (Schiff base formation; chemodosimeter)

A second important pattern is that aqueous compatibility varies systematically with mechanism type. Non-covalent VOC detection (PBSB) and the polarity-sensitive HCl probe (H3) operate in the solid or vapour phase, circumventing solvent compatibility challenges entirely. In contrast, reaction-based probes such as HNA and DTH still require mixed organic–aqueous buffers. The ratiometric formaldehyde probe (quinolimide-NBD) is a partial exception, operating in mixed solvent but providing a built-in intensity correction that reduces artefactual interference. Only Bis-TPI and DTH have demonstrated real sample applicability in agricultural matrices and cell imaging respectively. Most of the remaining probes in this table remain at the proof-of-concept level without real environmental or biological matrix validation.

A critical gap evident across Table 5 is the near-complete absence of probes for the most commercially significant pesticide classes such as organophosphates (chlorpyrifos, malathion), triazines (atrazine), and the globally prevalent glyphosate. These compounds represent far greater regulatory monitoring challenges than mesotrione or Starane.

This represents a practically important and largely unclaimed territory for Schiff base chemosensor design. The complementarity of the four formaldehyde probes (HBD: highest sensitivity; DTH: FA/AA discrimination; NapNH_2_: dual analyte; quinolimide-NBD: wide linear range) suggests that the most analytically complete approach for any single neutral molecule class would be a sensor array combining probes with orthogonal response characteristics. However, this strategy has not yet been systematically explored for Schiff base-derived neutral organic sensing platforms.

### 2.5 Acid–base and redox-responsive detection of neutral inorganic species

While the preceding sections focused on ionic and organic molecular analytes, Schiff base-derived fluorescent probes have also been developed for two inorganic species of considerable toxicological, industrial, and biomedical importance: hydrogen peroxide (H_2_O_2_) and hydrogen chloride (HCl) (Table 6). These analytes present fundamentally distinct detection challenges that require different mechanistic strategies. H_2_O_2_ is an oxidising reactive oxygen species (ROS) with dual biological roles: at low physiological concentrations (nanomolar range) it functions as an intracellular second messenger in redox signalling pathways, while at elevated levels it causes oxidative damage to proteins, lipids, and DNA that is implicated in cancer, cardiovascular disease, and neurodegeneration.^132,133^ Industrial relevance arises from its use in bleaching, disinfection, and chemical synthesis, where worker exposure monitoring requires sensitive vapour- and solution-phase detection. In contrast, HCl is a strong Brønsted acid that interacts with Schiff base probes through protonation of the imine nitrogen. This response provides a useful model for understanding the acid–base photophysics of the azomethine group in both polar and apolar media. The probes reviewed in this section; H1 and H2 for H_2_O_2_, and H3 for HCl; collectively illustrate how Schiff base scaffolds can be engineered to respond selectively to both oxidative and protonation-driven analytes through distinct electronic pathways.

**Table 6:** Comparative summary of Schiff base-based probes for neutral inorganic species (H_2_O_2_ and HCl)

Probe	Analyte	Detection strategy	Mechanism	LOD	Stoichiometry	Key strength	Key limitation	Reversibility/reusability
H1 (ref. 134)	H_2_O_2_	Fluorescence	Phenothiazine sulphur oxidation by H_2_O_2_; D–A balance disruption; fluorescence quenching	8.4 × 10^−7^ M	1 : 2 (probe : H_2_O_2_)	Sub-µM LOD; white-light dual function; high binding constant; DFT + NMR validated	No live-cell imaging; turn-off mode; ionic liquid medium limits broad application	Likely irreversible (sulphur oxidation; not demonstrated)
H2 (ref. 134)	H_2_O_2_	Fluorescence	Phenothiazine sulphur oxidation; modified D–A character; emission quenching	1.96 × 10^−7^ M	1 : 2 (probe : H_2_O_2_)	Lower LOD than H1; sub-µM detection; white-light emission retained until analyte threshold	No biological validation; turn-off; selectivity in complex ROS mixtures not fully reported	Likely irreversible (sulphur oxidation; not demonstrated)
H3 (ref. 135)	HCl	Dual-mode (colorimetric + fluorescence)	Imine nitrogen protonation (–C=N– → iminium); HOMO–LUMO gap narrowing; CT/LE/PT emission modulation	Not quantified	2 : 1 (fluorescence); 1 : 1 (UV-vis)	X-ray structural proof; 4-scale solvatochromism; naked-eye colorimetric response; broad mechanistic insight	Strong solvent dependence; no aqueous or biological validation; LOD not reported	Reversible in principle (protonation-based; solvent-dependent)

**Fig. 24:**
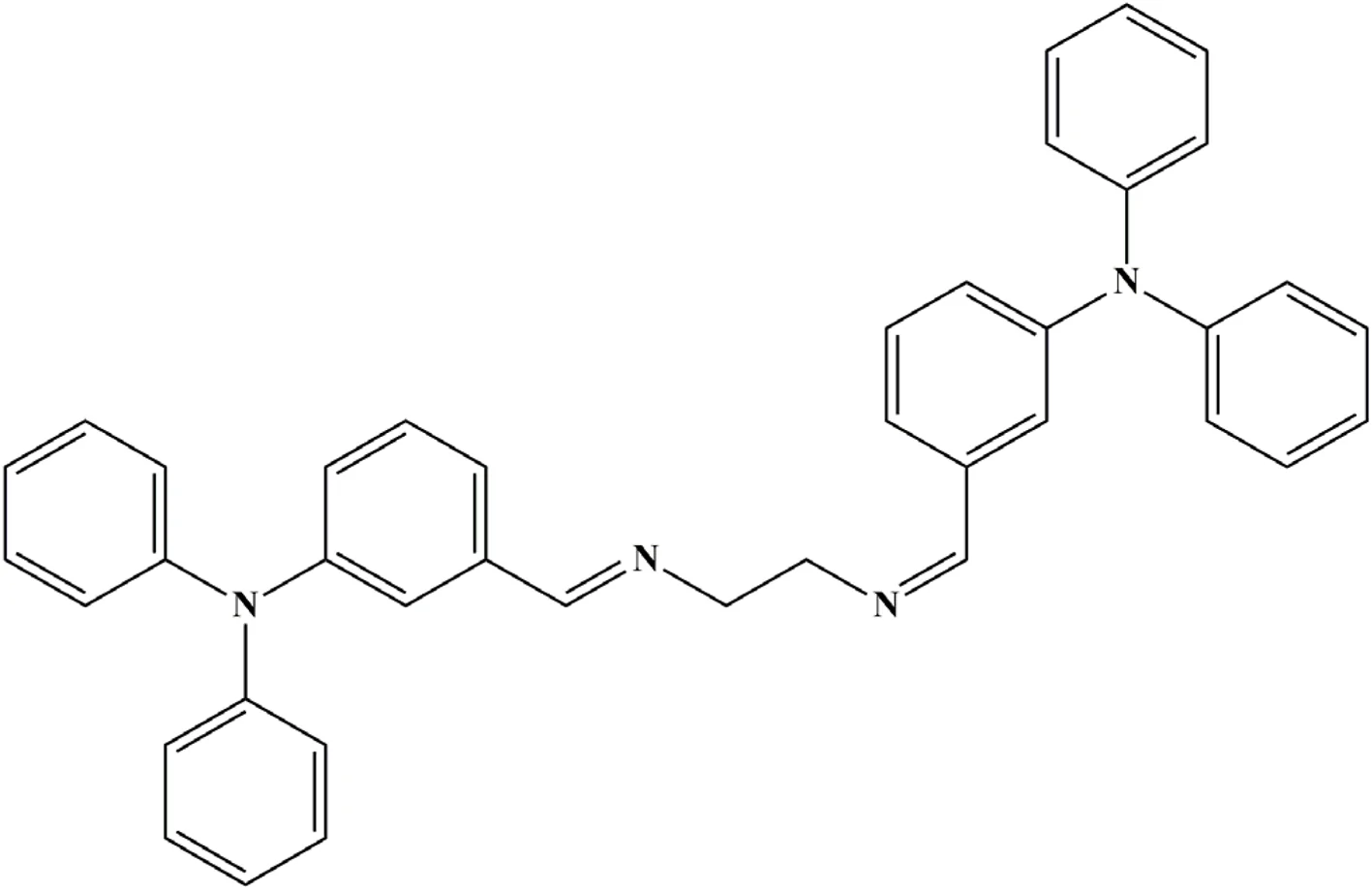
Structure of triphenylamine-based bis-Schiff base probe H1 for selective H_2_O_2_ detection. Co-formulation with rhodamine B generates white-light emission in ionic liquid media; H_2_O_2_ oxidises the donor centre, quenching emission selectively over other ROS (LOD 8.4 × 10^−7^ M; binding constant 9.90 × 10^9^ M^−1^); 1 : 2 stoichiometry confirmed by Job's plot; imine bond participation confirmed by ^1^H NMR titration and DFT.

**Fig. 25:**
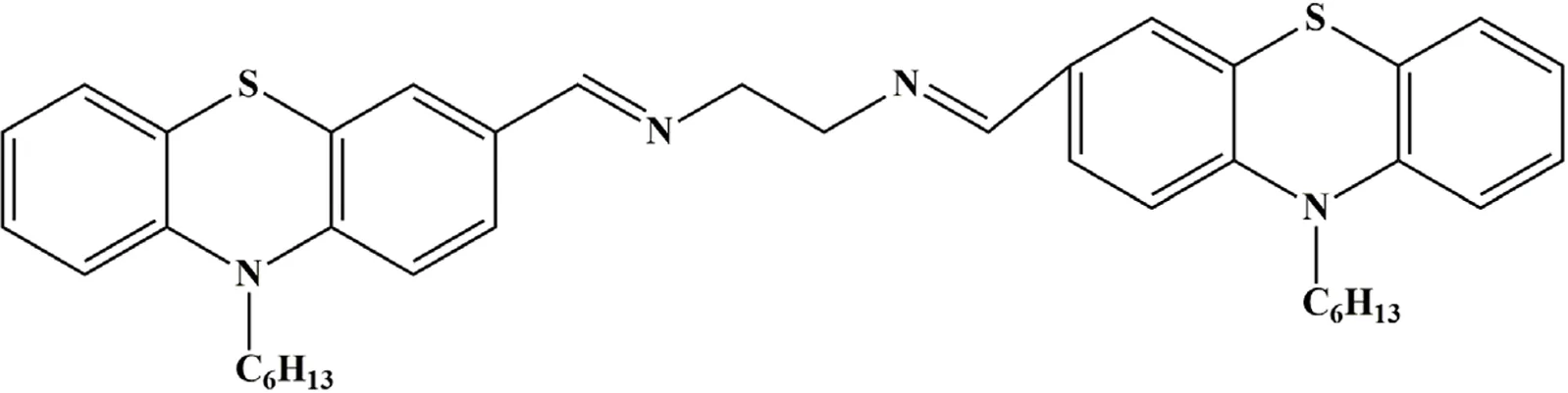
Structure of bis-phenothiazine-based Schiff base probe H2 for selective H_2_O_2_ detection. H_2_O_2_ oxidises the phenothiazine sulphur centre, quenching donor emission selectively over other ROS (LOD 1.96 × 10^−7^ M; binding constant 1.64 × 10^9^ M^−1^); 1 : 2 stoichiometry confirmed by Job's plot; imine bond participation confirmed by ^1^H NMR titration and DFT.

Barot *et al.* synthesised two phenothiazine–triphenylamine Schiff bases, H1 and H2, which, when co-formulated with rhodamine B dye in ionic liquid media, exhibited strong white-light emission through additive colour mixing of the individual emission components.^134^ The Schiff base structures provide the blue-green fluorescent donor component of the white-light system, while rhodamine B contributes the red emission component; together they span the visible spectrum to produce a Commission Internationale de l’Éclairage (CIE) white-light coordinate. H_2_O_2_ sensing operates through selective oxidation of the electron-rich phenothiazine sulphur centre by H_2_O_2_, forming the sulphoxide product that perturbs the donor–acceptor electronic balance of the Schiff base scaffold, thereby quenching the phenothiazine-based emission with high selectivity over other ROS including ˙OH, and ClO^−^. Both H1 and H2, whose structures are shown in Fig. 24 and 25 respectively, exhibited 1 : 2 (probe : H_2_O_2_) stoichiometric binding confirmed by Job's plot, with exceptionally low LODs of 8.4 × 10^−7^ M (H1) and 1.96 × 10^−7^ M (H2), and very high binding constants of 9.90 × 10^9^ M^−1^ and 1.64 × 10^9^ M^−1^ respectively. DFT calculations demonstrated that the HOMO–LUMO energy gap follows the trend H1 > H2 > rhodamine B, and decreases markedly upon H_2_O_2_ complex formation, consistent with ground-state electronic stabilisation of the oxidised adduct. ^1^H NMR titration studies confirmed involvement of the azomethine C=N bond in the probe–analyte interaction, with diagnostic shifts in the imine proton signal upon H_2_O_2_ addition. Analytically, the sub-micromolar LODs of H1 and H2 are among the most sensitive reported for Schiff base-based H_2_O_2_ probes. These values are well below the physiologically relevant intracellular H_2_O_2_ concentration range (∼0.1–1.0 µM), which is necessary for reliable detection of basal redox signalling. The dual functionality as both white-light emitters and ROS sensors is architecturally distinctive, as most fluorescent probes sacrifice white-light capability upon analyte sensing. The primary limitation is the absence of live-cell imaging validation, which would confirm intracellular biocompatibility and membrane permeability essential for ROS monitoring in physiologically relevant contexts.

**Fig. 26:**
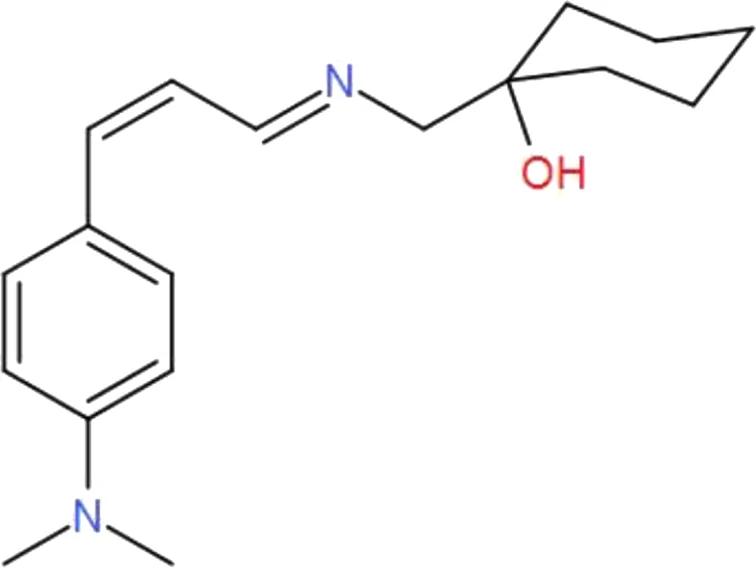
Schiff base probe H3 for HCl detection *via* imine nitrogen protonation. HCl protonates the C=N nitrogen to form the iminium cation, lowering the HOMO–LUMO gap and inducing yellow-to-deep-red photocoloration with a bathochromic fluorescence shift from 430 nm to 480 nm; 2 : 1 and 1 : 1 stoichiometries observed; X-ray crystallography confirms C=N bond as primary binding site; Stokes shift range 1789–7405 cm^−1^ across solvents (Lippert–Mataga, Kamlet–Taft, Catalán, Laurence scales); dipole moment change Δ*µ* = 11.9 D; three emission modes (CT, LE, PT) identified.

The photochemistry of Schiff base probes under strong acid exposure represents a mechanistically rich but analytically underexplored domain. Jiménez-Sánchez and Santillan reported that the Schiff base probe H3 undergoes a rapid photocoloration response upon exposure to HCl. The probe changes from yellow to deep red, accompanied by a bathochromic fluorescence shift from 430 to 480 nm and significant fluorescence quenching (Fig. 26).^135^ This response arises from protonation of the imine nitrogen, the most basic site in the Schiff base structure. Formation of the iminium cation (–CH=N^+^H–) lowers the HOMO–LUMO energy gap, shifts the absorption band into the visible region, and produces the characteristic yellow-to-deep-red colour change. Binding stoichiometries of both 2 : 1 and 1 : 1 (H3 : HCl) were detected by fluorescence and UV-visible spectroscopy respectively, reflecting the presence of two distinct HCl-responsive sites with different binding affinities within the molecular scaffold. X-ray crystallography provided definitive structural evidence that the C=N linkage is the primary site of HCl interaction, establishing unambiguous mechanistic clarity that is absent from many acid-responsive fluorescent probe reports which rely solely on solution spectroscopic evidence.

A particularly thorough aspect of the H3 study is the systematic solvatochromic characterisation using four independent polarity scales: Lippert–Mataga, Kamlet–Taft, Catalán, and Laurence. This multi-framework approach, which is rarely applied to Schiff base probes, revealed consistent positive solvatochromism across all four polarity scales. The Stokes shift increased from 1789 cm^−1^ in water to 7405 cm^−1^ in benzyl alcohol, confirming that the excited state is substantially more polar than the ground state. Three distinct emission modes were identified: charge-transfer (CT) emission, locally excited (LE) emission, and proton-transfer-mediated (PT) emission, which interconvert as a function of solvent polarity and HCl concentration. The computed dipole moment change of 11.9 D upon photoexcitation, among the highest reported for Schiff base fluorophores, confirms the strongly charge-transfer character of the emissive state and explains the exceptional solvatochromic range. Although these mechanistic insights distinguish H3 as one of the most comprehensively characterised Schiff base probes, its practical analytical application is limited by the strong solvent dependence of its sensing response. Consequently, quantitative HCl determination requires careful solvent control and calibration for each application medium. The absence of sensing data in aqueous conditions specifically limits utility for biological HCl monitoring (*e.g.*, gastric acid sensing), which would represent the most significant translational application of this probe class.

The three probes reviewed cover analytically important inorganic targets but differ substantially in their translational maturity. The H1 and H2 probes represent a mechanistically sound and practically credible approach to H_2_O_2_ sensing, with sub-micromolar LODs that are analytically relevant for both biological ROS monitoring and industrial process control. The phenothiazine oxidation mechanism provides inherent selectivity for H_2_O_2_ over other ROS because the sulphur oxidation potential of phenothiazine is thermodynamically matched to H_2_O_2_ but not to weaker oxidants. However, the mechanistic basis for the reported binding constants (approximately 10^9^ M^−1^) is not fully rationalised. Values of this magnitude are unusual for non-covalent probe–analyte interactions and may instead reflect irreversible oxidation chemistry rather than true equilibrium binding. If so, the probes would be unsuitable for reversible or continuous monitoring. This ambiguity regarding reversibility represents the most critical unresolved mechanistic question for H1 and H2.

The H3 probe for HCl is distinguished by its exceptional photophysical characterisation. It is arguably the most thoroughly characterised solvatochromic Schiff base probe identified in this review. The four-scale polarity analysis, X-ray crystallographic confirmation of the binding site, and identification of three distinct emission modes provide a level of mechanistic rigour that most Schiff base sensing studies do not approach. However, from an analytical standpoint, the absence of a quantitative LOD, the strong solvent-dependence of the sensing response, and the lack of aqueous matrix applicability represent substantial practical limitations that prevent immediate deployment. Unlike the oxidation mechanism of H1/H2, the protonation-based HCl sensing mechanism is fundamentally reversible. This reversibility could potentially be exploited for continuous HCl vapour monitoring in industrial environments, although this application was not explored in the original report. A solid-state film format of H3, analogous to the PBSB VOC sensor reviewed, would represent a straightforward translational advance that leverages the strong solvatochromic properties of this probe for portable industrial HCl detection.

Comparison of H1/H2 (H_2_O_2_) and H3 (HCl) highlights two contrasting strategies for inorganic sensing with Schiff bases. Oxidation-driven ROS sensing achieves excellent sensitivity but is likely to be irreversible, whereas protonation-driven acid sensing offers mechanistic richness and potential reversibility but remains limited by solvent dependence and incomplete analytical validation. The most practically impactful future direction for both probe types is integration into solid-state or vapour-responsive formats that circumvent solvent compatibility constraints, combined with real biological or industrial matrix validation to bridge the gap between photophysical characterisation and deployable analytical tools.

## 3 Applications of Schiff base chemosensors

**Fig. 27:**
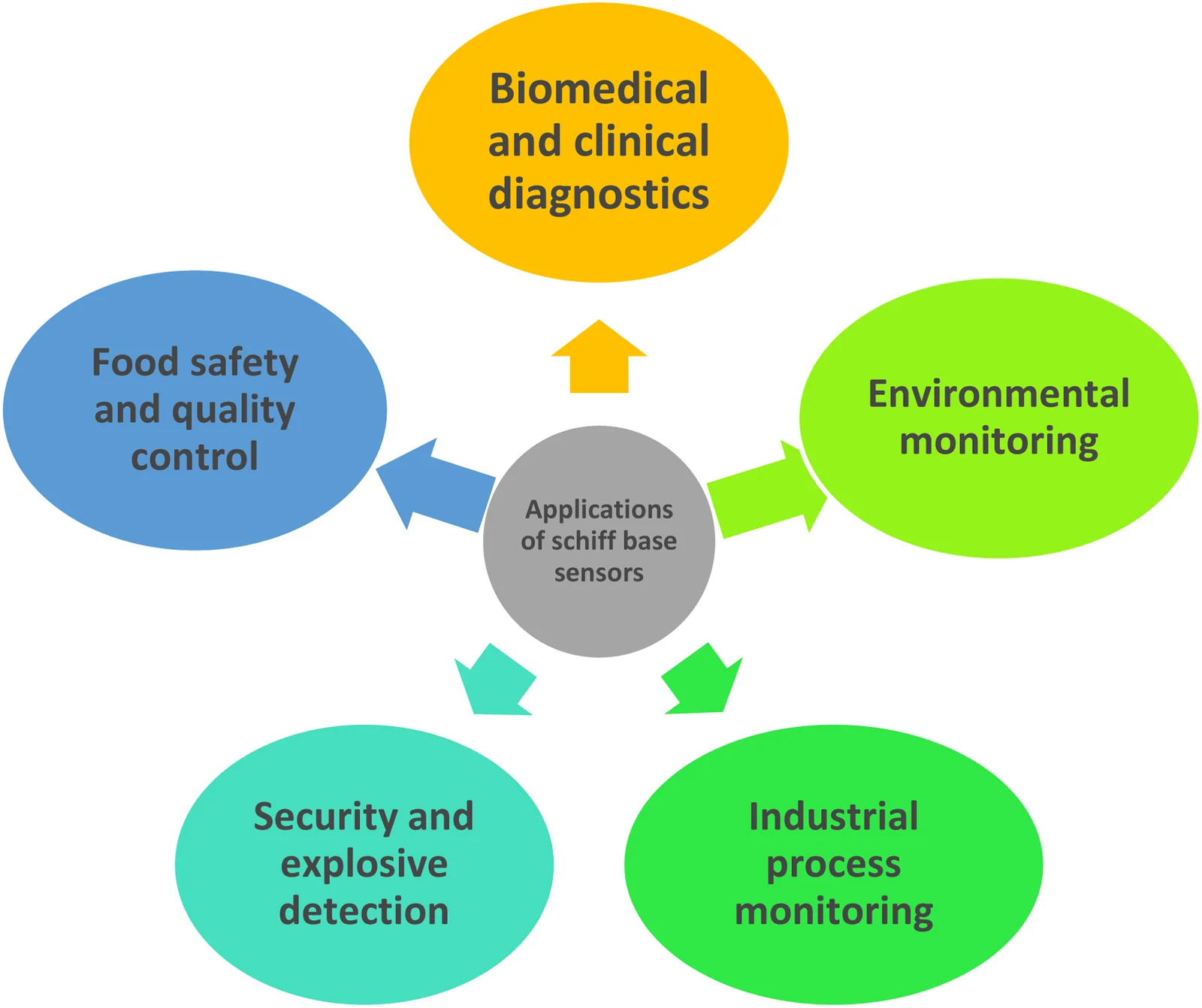
Schematic overview of the five principal application domains for Schiff base-based chemosensors: biomedical and clinical diagnostics, environmental monitoring, industrial process monitoring, security and explosive detection, and food safety and quality control.

The diverse sensing capabilities of Schiff base chemosensors reviewed in the preceding sections translate into a wide range of practical applications, summarised schematically in Fig. 27. Schiff base chemosensors have shown significant promise in clinical diagnostics due to their high sensitivity and compatibility with biological systems.^136–138^ These probes have been effectively utilised for the detection of disease-related biomarkers such as amino acids, proteins, reactive oxygen species, and aldehydes in biological fluids.^139–142^ Amino acids including arginine, cysteine, histidine, and selenocysteine play essential roles in wound healing, cell division, antioxidant defence, and immune function, and their probe-based detection in urine and blood samples, as demonstrated by probes S4 (histidine in urine), S5 (vitamin B12 in blood and urine), and NPC (HSA in urine), establishes a pathway toward point-of-care clinical diagnostics.^139,143^ The application of Schiff base probes to live-cell imaging, demonstrated by MC-Sec (selenocysteine), MCAs (VDPs), TPE-DPP (GSH), and the hydrazine probes S7 and S8, enables real-time monitoring of intracellular biochemical processes and offers valuable insights for early disease diagnosis.^144,145^

Environmental monitoring represents a major application domain. Probes reviewed in Sections 2.3–3.4 have been applied to the detection of hydrazine, nitroaromatic explosives, herbicides, and chlorinated VOCs in water, air, and soil matrices, as illustrated in Fig. 27.^146,147^ Particularly notable is the vapour-phase detection demonstrated by the solid-state probes S6, S7, S8 (hydrazine), and PBSB (chlorinated VOCs), which extend the analytical reach of Schiff base chemistry from solution-phase laboratory assays toward portable field-deployable formats. In the food safety domain, Schiff base chemosensors have been applied to the detection of formaldehyde, biogenic amines, and spoilage indicators, with the Bis-TPI probe validated in fresh leaf samples, S6 (vitamin B1) validated in peanuts and tablets, and the quinolimide FA probe covering the high-concentration range relevant to food adulteration monitoring.^148–150^

For security and defence applications, Schiff base sensors including CCH (*p*-nitrotoluene, 25 nM LOD) and DPTZ-SB (picric acid, 0.2 ppb with paper-strip smartphone readout) demonstrate analytical performance approaching or exceeding regulatory thresholds for explosive detection and shown in Fig. 27.^151,152^ In industrial process monitoring, the PBSB/SPR platform and Barot *et al.*’s^134^ H_2_O_2_ probes (H1 and H2) exemplify applications for monitoring hazardous intermediates and reactive oxygen species in process streams.^153^ The most advanced practical deployment format demonstrated in this review is the DPTZ-SB paper strip with smartphone readout, which integrates Schiff base chemistry into a user-friendly, instrument-free point-of-care device architecture. When coupled with smartphone-based colorimetric analysis, as demonstrated by Zhang *et al.*^154^ (visual sensor array for aromatic amines),^123,155^ these portable platforms offer cost-effective and field-deployable solutions that represent the translational frontier of Schiff base chemosensor research.^156^

## 4 Critical assessment of Schiff base chemosensor platforms

A systematic survey of the Schiff base chemosensor literature across ten analyte categories reveals a field of considerable mechanistic breadth but uneven translational maturity. The azomethine scaffold supports at least eight distinct photophysical transduction pathways, namely PET, ICT, ESIPT, CHEF, FRET, AIEE, p*K*_a_ shift, and direct imine bond cleavage. Many probes combine two or more of these mechanisms within a single molecular architecture.^55,62^ This mechanistic versatility is the defining analytical strength of Schiff base probes and is unmatched by single-mechanism fluorophore platforms such as rhodamine (predominantly ring-opening) or BODIPY (predominantly PET).^69^ However, the mechanistic diversity evident across Tables 1–5 does not translate uniformly into analytical utility. Turn-off (quenching) responses dominate across the nitroaromatic, thiophenol, and neutral organic sensing categories, accounting for the majority of probes in these sections.^157^ Turn-off detection is inherently less reliable in complex environmental and biological matrices where background fluorescence, photobleaching, and probe concentration variability can generate false-positive signals that are analytically indistinguishable from genuine analyte responses. This fundamental limitation has been identified in every section of this review yet remains inadequately addressed in the field as a whole, with ratiometric or turn-on designs representing a minority of reported systems. The progression from single-intensity turn-off through turn-on to ratiometric detection represents the most consequential design frontier for the next generation of Schiff base probes across all analyte classes.

The single most pervasive structural liability identified across all ten analyte categories is the susceptibility of the C=N bond to hydrolysis under aqueous and physiologically relevant conditions.^58^ This limitation undermines sensing performance in three distinct ways. First, it reduces probe stability over the timescale of analysis. Second, it competitively consumes the recognition unit intended for the target analyte. Third, it introduces pH-dependent variability in fluorescence output that is difficult to deconvolute from genuine analyte responses. Probes that rely on imine nitrogen coordination as the primary binding event, including S4 (histidine),^72^ S6 (vitamin B1),^88^ and the SBB hydrazine probe,^101^ are particularly vulnerable. Strategies demonstrated to mitigate this limitation include the use of electron-withdrawing substituents on the imine-flanking aromatic ring to reduce imine nucleophilicity, macrocyclisation of the Schiff base to impose geometric rigidity as demonstrated in Bis-TPI for starane detection,^158^ immobilisation within a crystalline MOF framework as shown for the TNP sensor,^110^ and reaction-based chemodosimetric designs in which the imine is consumed rather than preserved during sensing, as in HNA for mesotrione and TPE-DPP for GSH.^82,159^ Among these strategies, MOF immobilisation and reaction-based mechanisms provide the most robust aqueous stability, but at the cost of single-use irreversibility. A comprehensive synthetic approach combining macrocyclisation with an electron-withdrawing substituent pattern has not yet been systematically explored for the probe classes reviewed here and represents a high-priority synthetic direction.

A cross-category analysis of solvent systems reported in Tables 1–5 reveals that dependence on mixed organic-aqueous co-solvents (typically DMSO–water, DMF–water, or acetonitrile–water at organic fractions of 20 to 60%) is the dominant practical limitation affecting translational applicability. Of the solution-phase probes reviewed, only a minority operate in predominantly aqueous or fully aqueous media without significant organic co-solvent requirements. These include TBA (hydrazine),^102^ TPE-DPP (GSH),^82^ DPTZ-SB (picric acid),^160^ Bis-TPI (starane),^158^ HBD (formaldehyde),^161^ and NapNH_2_ (formaldehyde and phosgene).^161^ This solvent dependence is particularly consequential for biologically targeted probes, as the cytotoxic effects of DMSO and DMF at working concentrations preclude their direct use in live-cell assays and would complicate clinical translation. Real-sample validation is demonstrated for fewer than half the probes reviewed. TBA and SBB were tested in environmental river and tap water samples,^101,102^ S4 was applied to urine and cell imaging,^72^ S5 to blood and urine,^86^ DPTZ-SB to real water *via* paper strip,^160^ and Bis-TPI to agricultural water and fresh leaf samples.^158^ The remaining systems are validated exclusively in spiked laboratory solutions prepared in organic-aqueous buffers, which bears limited relevance to the complex matrix environments of genuine analytical deployment. Bridging this validation gap, from laboratory proof-of-concept to performance characterisation in real biological fluids, environmental samples, and food matrices, is the most urgent translational requirement across all analyte categories reviewed.

Selectivity assessment across the reviewed systems reveals a field-wide tendency toward validation against a narrow panel of structurally similar competitors rather than against the full diversity of interfering species encountered in realistic sample matrices. Arginine probes S1 and S2 demonstrate selectivity over standard amino acid panels but are not tested in the presence of competing guanidinium-containing metabolites at physiologically realistic concentrations. Thiophenol probe SBMD shows selectivity over aliphatic thiols at equimolar concentrations, but the competitive interference from H_2_S and polysulfide species, which are abundant in biological and environmental matrices and are chemically competent for SNAr at the DNBS recognition unit, has not been rigorously characterised. Nitroaromatic probes generally rely on π–π stacking and electron-transfer quenching to discriminate among structurally related analytes. However, most were evaluated only against selected nitroaromatic congeners rather than the broader range of electron-deficient compounds encountered in contaminated matrices.^110,160^ This pattern of narrow interference testing is a systemic limitation that, if identified during peer review, would represent a significant criticism for high-impact journal submission. Future probe evaluation protocols should adopt standardised interference panels that include biologically and environmentally relevant competitors at concentration levels reflecting actual co-occurrence ratios rather than equimolar spike conditions.

A critical functional limitation that distinguishes laboratory demonstrations from deployable analytical tools is reversibility. Of the probes reviewed across all analyte categories, reversible binding, where the original fluorescence response is quantitatively restored upon removal of the analyte, is demonstrated for only a small minority, including the Cu^2+^-displacement systems (S3, S4),^69,72^ the PBSB VOC sensor (non-covalent van der Waals adsorption),^162^ and the H3 HCl probe (protonation-based).^163^ The majority of probes reviewed operate through irreversible covalent mechanisms. These include SNAr for SBMD (thiophenol),^97^ imine condensation for hydrazine probes,^101,102,104^ C=N cleavage for HNA and TPE-DPP,^159^ and oxidation for H1 and H2.^164^ Irreversible probes function as single-use chemodosimeters, suitable for endpoint detection but unable to be deployed in continuous monitoring formats, regenerated for reuse, or applied to kinetic studies of analyte fluctuations. For applications such as industrial process monitoring of H_2_O_2_ and chlorinated VOCs, environmental surveillance of hydrazine, and real-time intracellular imaging of GSH redox dynamics, reversibility is not merely desirable but analytically necessary. The design of reversible Schiff base probes for analytes currently addressed only by irreversible chemodosimetric mechanisms, particularly thiophenols, formaldehyde, and hydrazine, represents a high-priority design challenge whose solution would substantially elevate the translational value of this scaffold class.

Viewed collectively, the Schiff base chemosensor literature reviewed here is at an inflection point between molecularly sophisticated proof-of-concept chemistry and analytically deployable tools. The structural and mechanistic foundations are well-established; the azomethine scaffold can be tuned to address an exceptionally diverse analyte landscape with detection limits that are, in the majority of cases, already analytically sufficient for practical applications. The primary bottlenecks are selectivity, matrix compatibility, reversibility, and device integration rather than sensitivity. The most promising trajectories identified across the full analyte landscape are as follows. First, the AIEE-based solid-state probe architecture exemplified by S7, S8, and DPTZ-SB simultaneously addresses aqueous compatibility, vapour-phase detection, and solid-state device integration. Second, the reaction-based selectivity-by-reactivity approach of MC-Sec and SBMD achieves analyte discrimination through intrinsic chemical reactivity differences rather than fragile thermodynamic binding preferences. Third, the MOF-based platform of the TNP sensor addresses imine hydrolysis through structural immobilisation while amplifying sensitivity through the extended porous sensing framework. Developing a new generation of fully aqueous-compatible, ratiometric, reversible, and device-integrated Schiff base chemosensors will require close integration of synthetic chemistry, materials science, computational probe design, and regulatory-aligned analytical validation. Given the rapid progress across these areas, the field is well positioned to achieve this goal within the coming decade.

Beyond the probe-level limitations discussed above, three systemic barriers to real-world deployment of Schiff base chemosensors warrant explicit discussion. First, long-term stability remains largely uncharacterised across the reviewed probe systems. The imine (C=N) bond, which is the defining structural feature of all Schiff base probes, is susceptible to hydrolysis under prolonged aqueous exposure, acidic conditions, and elevated temperatures, all of which are routinely encountered in field deployment scenarios. Photobleaching under continuous excitation further erodes analytical performance over extended monitoring periods. Of the probes reviewed, only the MOF-based TNP sensor^110^ and the solid-state AIEEgen probes S7/S8 (ref. 106) have demonstrated structural robustness under aqueous conditions, while long-term stability data under storage and repeated use conditions are absent for the majority of systems including DPTZ-SB^160^ and PBSB.^162^ Second, interference from complex matrices represents a critical and systematically under characterised limitation. Real biological fluids such as blood, urine, and cell lysates contain high concentrations of competing thiols, metal ions, proteins, and pH buffers that can modulate probe fluorescence independently of the target analyte. Environmental water samples introduce humic acids, suspended particulates, and variable ionic strength that alter aggregation behaviour of AIEE-based probes and inner filter effects for turn-off systems. The narrow interference panels used in most reported studies, typically comprising fewer than ten structurally related competitors at equimolar concentrations, do not adequately reflect these matrix complexities. Third, device fabrication constraints remain a significant bottleneck for translation from laboratory proof-of-concept to field-deployable analytical tools. The transition from solution-phase sensing to solid-state formats requires optimisation of probe loading density, substrate porosity, optical readout geometry, and environmental sealing, none of which have been systematically addressed for the majority of Schiff base probes reviewed. DPTZ-SB^160^ and PBSB^162^ represent the most advanced current examples of device integration, but neither reports quantitative device stability, shelf life, or performance under field humidity and temperature extremes. Addressing these three barriers through standardised stability protocols, matrix-matched validation studies, and device engineering will be essential for advancing Schiff base chemosensors beyond laboratory demonstrations toward deployable analytical platforms.

## 5 Future scope

Computational approaches, including density functional theory (DFT), time-dependent DFT (TD-DFT), and molecular docking, represent underutilised but highly promising tools for the rational design of next-generation Schiff base chemosensors. DFT calculations can predict ground-state electronic properties, optimised molecular geometries, and frontier molecular orbital (HOMO–LUMO) energy gaps of candidate probe structures prior to synthesis, thereby narrowing the design space and reducing synthetic effort. TD-DFT extends this capability to excited-state properties, enabling prediction of absorption and emission wavelengths, Stokes shifts, and charge-transfer character that directly determine the photophysical sensing mechanism. Within this review, DFT calculations have already been employed to validate sensing mechanisms for probes TBA (hydrazine), SBB (hydrazine), CCH (*p*-nitrotoluene), and H1/H2 (H_2_O_2_), confirming their utility as mechanistic verification tools. However, their prospective application to guide probe design before synthesis remains rare in the Schiff base chemosensor literature. Molecular docking simulations offer a complementary capability for predicting probe–analyte binding geometries, interaction energies, and selectivity profiles, particularly valuable for probes targeting biological macromolecules such as HSA, VDPs, and enzyme-active-site analytes. Beyond these established computational methods, emerging machine learning and artificial intelligence frameworks trained on large fluorophore datasets have demonstrated the ability to predict emission wavelengths and quantum yields with high accuracy^165,166^ and recent AI-driven fluorescence probe discovery platforms have begun to accelerate the identification of novel sensing scaffolds.^167^ A systematic integration of these computational tools into the Schiff base probe design workflow, from initial scaffold screening through selectivity prediction to photophysical property optimisation, represents one of the highest-impact directions for the field and is strongly recommended as a priority for future investigations. The future development of Schiff base chemosensors must move beyond incremental optimisation of individual probe sensitivity toward addressing the systemic limitations identified throughout this review. The development of water-stable imine architectures through macrocyclisation, imine-to-amide conversion, or electron-withdrawing substituent strategies should be prioritised as a foundational materials challenge applicable across all analyte categories. Ratiometric probe designs, in which analyte recognition produces a shift in emission wavelength rather than a change in intensity, are needed to enable quantitative analysis in complex biological and environmental matrices where background variability obscures single-wavelength intensity responses; this is particularly urgent for the nitroaromatic and thiophenol sensing domains where all current Schiff base probes operate in turn-off mode.

Multiplexed sensing, the simultaneous detection of two or more analytes using a single probe or probe array, represents an underexplored but practically transformative direction. Early examples reviewed here—including CCH, which detects both Cu^2+^ and *p*-nitrotoluene, and NapNH_2_, which detects both formaldehyde and phosgene—demonstrate proof of principle. However, the deliberate design of Schiff base probes for dual or triple analyte discrimination using spectrally resolved channels has not yet been achieved. The integration of Schiff base sensing elements with nanomaterials including metal–organic frameworks (as demonstrated for TNP sensing), AIEE nanoparticles, electrospun polymer fibres, and molecularly imprinted polymer matrices offers routes to combine the molecular selectivity of the azomethine recognition unit with the signal amplification and structural stability of nano/macroscopic platforms. Solid-state, paper-based, and smartphone-readable sensor formats, demonstrated at proof-of-concept level by DPTZ-SB and PBSB in this review, must be further developed with long-term stability data, environmental durability testing, and regulatory-compliant validation before practical deployment.^168–170^

Translational readiness requires explicit attention to *in vivo* validation, pharmacokinetic profiling, and toxicity assessment for probes targeting biological analytes. Of the cellular imaging-capable probes reviewed, few have progressed beyond cell-line studies to tissue imaging or animal model validation. Translating laboratory fluorescent sensors into clinically deployable diagnostic reagents requires demonstration of biocompatibility in primary cell systems, ADME profiling, and eventual regulatory evaluation. None of these requirements has yet been addressed for the Schiff base probes reviewed here. This gap between laboratory demonstration and clinical translation remains the widest and most important frontier in the field. Finally, portable point-of-care integration, including microfluidic paper-based analytical devices (microPADs) with smartphone-based colorimetric readout, represents a practically achievable near-term goal that would transform several of the probes reviewed from laboratory curiosities into deployable public health tools.

An emerging and rapidly expanding frontier that is not yet reflected in the Schiff base sensing literature but offers transformative potential is the integration of artificial intelligence (AI) and machine learning (ML) with fluorescent probe design and data analysis. The development of fluorescent probes has historically been governed by trial-and-error synthetic exploration, guided by chemical intuition and empirical photophysical measurement. ML approaches are beginning to replace this paradigm with data-driven predictive frameworks. Ju *et al.* demonstrated that gradient boosted regression models trained on molecular descriptor databases of over 3000 organic fluorophores can predict emission wavelengths and photoluminescence quantum yields with high accuracy, enabling virtual screening of probe candidates before synthesis.^165^ Applied to Schiff base probes, such models could predict how azomethine substitution patterns, donor–acceptor configurations, and solvent effects modulate the ICT-driven emission wavelength, directly accelerating the rational design of probes targeting specific spectral windows. More recently, Zeng *et al.* introduced FLAME, an AI framework that integrates the largest open-source fluorophore database (FluoDB, 55 169 fluorophore–solvent pairs) with multiple prediction models and generative molecular design engines. This framework enables on-demand design of fluorophores with user-specified emission properties.^166^ The PROBY model of Jiang *et al.* extends this concept to target-specific probe discovery, predicting not only photophysical properties but also binding selectivity against specific biological targets from molecular structure alone.^167^

For colorimetric and smartphone-based sensing arrays, convolutional neural network (CNN) image analysis of colour responses enables quantitative analyte concentration determination from simple photograph inputs, removing the need for spectrophotometric equipment and enabling true point-of-care deployment of Schiff base colorimetric probes. Sensor array data from multiple Schiff base probes with overlapping selectivity patterns can be processed by principal component analysis (PCA) or linear discriminant analysis (LDA) to discriminate analytes that no individual probe can resolve alone, a strategy that is particularly promising for the nitroaromatic analyte class where selectivity among structurally similar compounds remains poor. QSAR models specifically trained on Schiff base fluorophore datasets, which do not yet exist in the literature, would enable prediction of how imine bond geometry, donor–acceptor substituent effects, and macrocyclic framework modifications affect sensing performance metrics including LOD, selectivity coefficient, and response time. The rapid development of AI tools for fluorescent probe design, together with the structural modularity of Schiff base chemistry, makes these sensors an ideal platform for ML-guided molecular engineering. This convergence is likely to become one of the most impactful research directions for the field over the coming decade.

## 6 Conclusion

This review has systematically surveyed Schiff base-derived fluorescent and colorimetric chemosensors across ten analyte categories, spanning biologically relevant amino acids and proteins, vitamins, reactive small molecules (thiophenols and hydrazine), nitroaromatic compounds, herbicides, formaldehyde and aldehydes, chlorinated VOCs, and inorganic species including H_2_O_2_ and HCl. The collective findings establish that Schiff base scaffolds are capable of achieving analytically competitive performance across a remarkable breadth of target analytes, with detection limits spanning from the picomolar range (S5 for vitamin B12, LOD 1.18 × 10^−9^ M) to the sub-ppb level (DPTZ-SB for picric acid, LOD 0.2 ppb) and the sub-ppb vapour range (S7/S8 for hydrazine, LOD 0.4–0.5 ppb). The mechanistic diversity operative within Schiff base sensing frameworks is particularly notable. PET, ICT, ESIPT, CHEF, FRET, AIEE, p*K*_a_ shift, and direct imine bond cleavage have all been demonstrated as viable transduction pathways within this single ligand class, and in several instances multiple mechanisms operate synergistically within a single probe architecture (TPE-DPP combining AIEE and Schiff base reactivity; PBAS combining AIEE and ESIPT; CDB-Am combining *in situ* Schiff base formation with PET suppression and AIEE). This mechanistic versatility is the defining analytical strength of the azomethine functional group and distinguishes Schiff bases from single-mechanism fluorophore platforms. However, imine bond hydrolysis under aqueous or acidic conditions remains the single most persistent structural liability across the entire probe landscape reviewed, compromising sensor stability in physiologically and environmentally relevant matrices and affecting those probes that rely on coordination through the imine nitrogen as the primary binding event. This limitation was identified across sections covering metal ion sensing, thiol sensing, and formaldehyde detection, and represents the primary structural challenge for the next generation of Schiff base probe design. The field's primary constraints, consistently evident across all analyte categories, lie in selectivity, aqueous operability, reversibility, and real-sample validation rather than in analytical sensitivity, which is generally already sufficient for practical applications. Dependence on mixed organic–aqueous solvents is the most pervasive practical limitation, appearing in probes for amino acids (S1, S3), hydrazine (SBB, TBA, TPA/TPB), and nitroaromatics (CCH, SBZC). Irreversible response mechanisms, inherent to reaction-based chemodosimetric probes, prevent sensor reuse and limit deployment in continuous monitoring applications. The most promising recent developments toward addressing these constraints include the AIEE-based solid-state probes S7 and S8 (aqueous-compatible, multi-matrix validated), the MOF-based TNP sensor (water-stable, structurally prevents imine hydrolysis), the DPTZ-SB paper-strip platform (portable, smartphone-readable), and the p*K*_a_ shift-based MC-Sec probe (intrinsic selectivity by reactivity). Continued advances in structure–property understanding, computational probe design, materials science, and digital sensing technologies will accelerate the development of Schiff base chemosensors. These advances should enable their evolution into robust, field-ready platforms for diagnostics, environmental surveillance, food safety, and security monitoring.

## Conflicts of interest

The authors affirm that they do not possess any identifiable conflicting financial interests or personal ties that might have potentially influenced the findings provided in this paper.

## Data Availability

This review article does not report any new experimental data. All information discussed is derived from previously published studies, which have been cited in the text. While preparing this work, the author(s) used Claude to improve the readability and language of the manuscript. After using this tool/service, the authors reviewed and edited the content as needed and take full responsibility for the content of the manuscript.
